# Mapping the current landscape of locoregional therapy de-escalation trials in early breast cancer: a systematic review

**DOI:** 10.1038/s41523-025-00744-9

**Published:** 2025-03-30

**Authors:** Alan D. McCrorie, Hilary Stobart, David Dodwell, Stuart A. McIntosh, Shelley Potter

**Affiliations:** 1 The Patrick G Johnston Centre for Cancer Research, Queen’s University Belfast, Belfast, UK; 2 Independent Cancer Patients’ Voice, Cambridge, UK; 3 Oxford Population Health, University of Oxford, Oxford, UK; 4 Bristol Surgical and Perioperative Care Complex Intervention Collaboration, Translational Health Sciences, Bristol Medical School, Bristol, UK; 5 Bristol Breast Cancer Centre, North Bristol NHS Trust, Bristol, UK

**Keywords:** Breast cancer, Oncology, Medical research, Outcomes research, Clinical trial design

## Abstract

A systematic review undertaken to map the current landscape of locoregional de-escalation trials to inform future research. Online databases and trial registries were searched to identify ongoing, recently completed or published studies de-escalating surgery or radiotherapy in patients with early breast cancer. 97 trials evaluated de-escalation of surgery or radiotherapy in up to 94,866 participants. Surgery studies more commonly evaluated treatment omission/reduction after neoadjuvant systemic therapy (NST) and de-escalation of nodal treatment. Radiotherapy studies were more frequently biomarker stratified. Patients were rarely involved in study design. Research questions focused on response-adjusted treatment after NST and omission/reduction of locoregional therapy in patients with low- or intermediate-risk disease. Significant duplication was identified with multiple studies addressing similar questions. This systematic review demonstrates that the current de-escalation portfolio is inefficient, lacks patient focus and needs improvement. An internationally collaborative approach using innovative study designs and patient partnership will be essential to address this.

## Introduction

In 2020, over 2.3 million patients worldwide were diagnosed with breast cancer^1^, and improving outcomes mean that many will be long-term survivors^2^. Current treatments for breast cancer include a combination of locoregional and systemic therapies with most patients having surgery ± radiotherapy together with chemotherapy, targeted treatments and endocrine therapy according to tumour subtype and disease stage.

There is increasing evidence that not all patients, particularly those with low or intermediate risk disease, benefit from all treatments that they currently receive. Most, if not all, however, will experience complications, side effects and other treatment-related toxicities, which can have profound and long-lasting impacts on their well-being and quality of life^3^. In addition to the personal costs, continued recommendation of treatments with marginal or no benefit has significant societal and global implications, diverting scarce healthcare resources from patients who would benefit from more intensive treatments^4,5^. Patients are strongly supportive of more personalised treatment approaches but emphasise that high quality research is needed to underpin any change in practice, and to demonstrate that proposed reductions in treatment are ‘safe’ and do not compromise oncological outcomes^3^. Well-designed, efficient and timely trials evaluating how to safely reduce or ‘de-escalate’ components of breast cancer treatment in appropriately selected patient groups are therefore a global research priority^6–8^.

Locoregional therapy with surgery and/or radiotherapy is the mainstay of treatment for early breast cancer and optimising these treatments will have the greatest potential benefit for the increasing numbers of patients who have low- or intermediate-risk disease. Understanding the current landscape of locoregional therapy de-escalation trials is essential to ensure that future studies are designed to meet the needs of patients and the breast cancer community, address research gaps, minimise duplication of effort and research waste and promote efficient and timely generation of high-quality data that can be rapidly translated into practice. This systematic review therefore aims to map the current landscape of ongoing and recently completed de-escalation trials to inform future research.

## Results

**Fig. 1:**
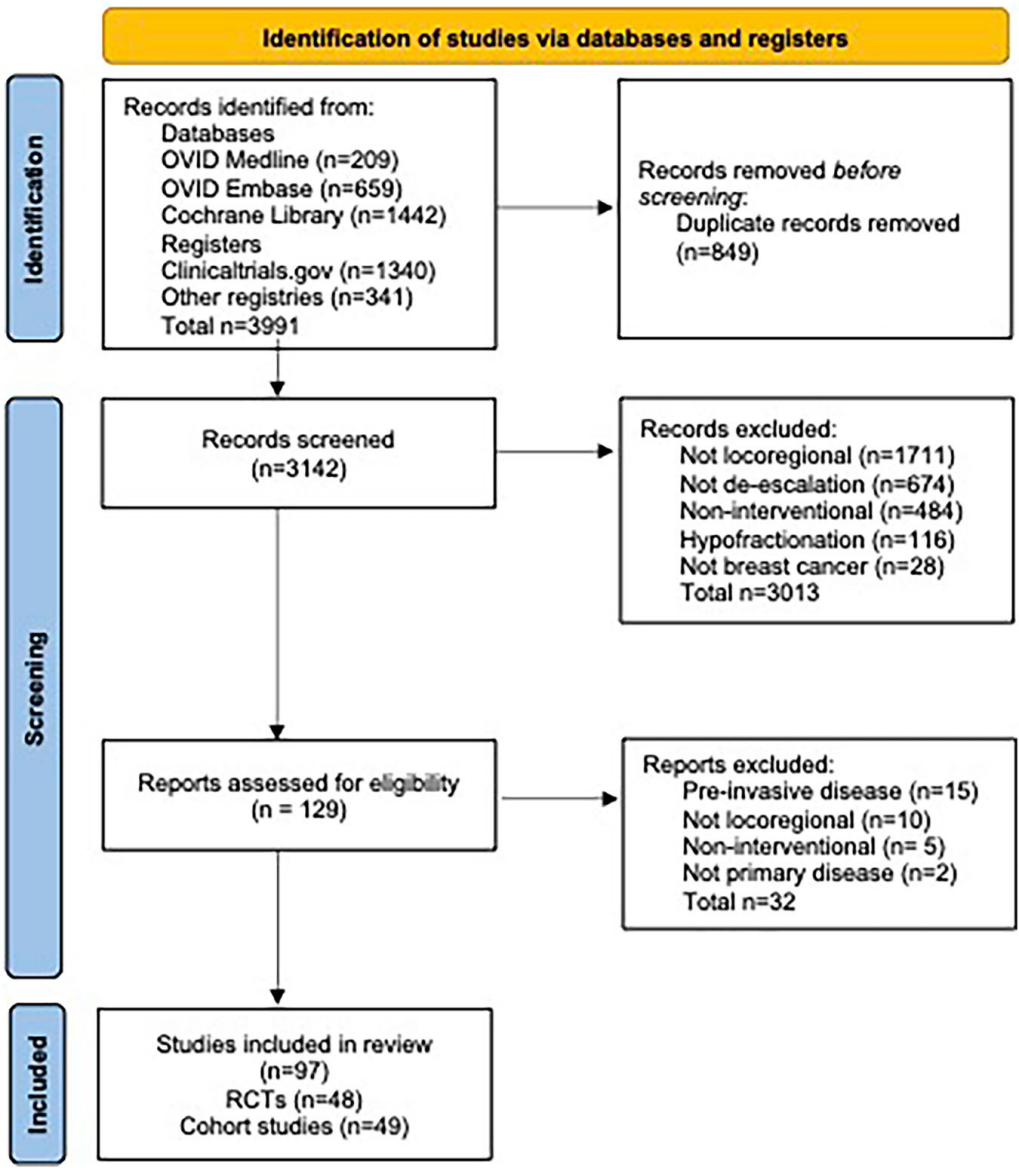
PRISMA diagram for the review.

The search identified 3991 results. After removal of duplicates, 3142 studies were screened for inclusion of which 129 were reviewed in full. 32 were excluded following discussion (Supplementary Table 1). A total of 97 studies (48 (49.5%) RCTs^9–56^ and 49 (50.5%) cohorts^57–105^) with 94,866 planned participants were included in the review (Fig. 1).

Characteristics of included studies are summarised in Table 1. Overall, most were multicentre (*n* = 67, 69.1%) and based in Europe or North America (*n* = 73, 75.3%). Of those reporting planned and actual recruitment numbers (*n* = 44), 61.4% (*n* = 27/44) achieved their accrual target, with only 8 (18.2%) closing due to recruitment issues. Recruitment periods ranged from 12 months to 12 years (median 5 years) with overall study durations of up to 27 years^78^ (median 9 years). Most studies (*n* = 77, 79.4%) had an oncological primary endpoint evaluated at a median of 60 months, but few studies (*n* = 9, 9.3%) selected participants based on biomarker stratification^19,22,26,52,66,75,79,90,91^. Patient-reported outcomes were only included as either a primary^17^ or co-primary^11,21^ endpoint in three (3.1%) studies and only 6 (6.2%)^11,48,51,53,55,91^ explicitly stated that patient advocates had been involved in study design. Details of included studies are summarised in Supplementary Table 2.

**Table 1:** Characteristics of included studies by study design and locoregional treatment modality de-escalated

	All surgery studies^c^ (*n* = 51)	All RT studies (*n* = 46)	*P* value^f^	All RCTs (n = 48)	All cohorts (*n* = 49)	*P* value^f^	All studies (*n* = 97)
No of centres							
Single centre	15 (29.4) 32	9 (19.6)	0.359	6 (12.5)	18 (36.7)	0.020	24 (24.7)
Multicentre	(62.8)	35 (76.1)		38 (79.2)	29 (59.2)		67 (69.1)
Unknown	4 (7.8)	2 (4.4)		4 (8.3)	2 (4.1)		6 (6.2
International^*^	8 (15.7)	16 (34.8)	0.030	18 (37.5)	6 (12.2)	0.004	24 (24.7)
Lead region							
Europe	26 (51.0)	23 (50.0)	0.139	30 (62.5)	19 (38.8)	0.139	49 (50.5)
North America	9 (17.6)	15 (32.6)		8 (16.7)	16 (32.7)		24 (24.7)
Asia	13 (25.5)	5 (10.9)		7 (14.6)	11 (22.5)		18 (18.6)
South America	2 (3.9)	1 (2.2)		2 (4.2)	1 (3.5)		3 (3.1)
ANZ	0 (0)	2 (4.4)		1 (2.1)	1 (2.0)		2 (2.1)
Africa	1 (2.0)	0 (0.0)		0 (0.0)	1 (2.0)		1 (1.3)
Year commenced							
Pre 2005	0 (0)	4 (8.7)	0.002	3 (6.3)	1 (2.0)	0.399	4 (4.1)
2005–2010	0 (0)	8 (17.4)		6 (12.5)	2 (4.1)		8 (8.3)
2011–2015	14 (27.5)	13 (28.3)		11 (22.9)	16 (32.7)		27 (27.8)
2016–2020	20 (39.2)	10 (21.7)		14 (29.2)	16 (32.7)		30 (30.9)
2021-date	17 (33.3)	11 (23.9)		14 (29.2)	14 (28.6)		28 (28.9)
Site de-escalated							
Breast	22 (43.1)	40 (87.0)	<0.001	26 (54.2)	36 (73.5)	0.048	62 (63.9)
Nodal areas	29 (56.9)	6 (13.0)		22 (45.8)	13 (26.5)		35 (36.1)
Biomarker stratified	1 (2.0)	8 (17.4)	0.009	4 (8.3)	5 (10.2)	0.751	9 (9.3)
NST	22 (43.1)	4 (8.7)	<0.001	8 (16.7)	18 (36.7)	0.026	26 (26.8)
Planned sample size (median, IQR, range)	340 (122–1560) (17–7095)	926 (208–1401) (25–4214)	0.086^e^	1386 (800–2134) (36–7095)	200 (100–385) (17–2400)	<0.001^e^	533 (170–1528) (17–7095)
Actual accrual (n = 46) (median, IQR, range)	847 (123–1818) (0–5505)	569 (200–1326) (12–4216)	0.872^e^	1396 (658–2074) (0–5505)	365 (194–596) (12–2400)	0.003^e^	634 (200–1688) (0–5505)
% of planned sample size recruited^a^ (median, IQR, range)	96.9 (56.7–100.1) (0–178.1)	100 (66.3–103.5) (15.4–204.2)	0.229^e^	85.7 (39.5–100.2) (0–137.7)	100.3 (100–124.7) (204.2-15.4)	0.006^e^	100 (58.8–102.7) (0–204.2)
Actual/planned recruitment (years, IQR, range)	4 (2–6) (1–8)	5 (4–7) (2–12)	0.004^e^	5 (4–7) (1–12)	4 (3–6) (1–11)	0.195^e^	5 (3–6) (1–12)
Overall duration of study (years, IQR, range)	8 (4–10) (1–25)	11.5 (8–15) (3–27)	<0.001^e^	11 (7–14.5) (1–25)	8 (5–11) (1–17)	0.013^e^	9 (7–9) (1–27)
Primary outcome^b^							
Locoregional recurrence	15 (29.4)	29 (63.0)	<0.001	18 (37.5)	26 (53.1)	0.067	44 (45.4)
Survival based	23 (45.1)	7 (15.2)		19 (39.6)	11 (22.5)		30 (30.9)
Other oncological	1 (1.2)	2 (4.4)		3 (6.3)	0 (0.0)		3 (3.1)
Patient reported	0 (0.0)	1 (2.1)		1 (2.1)	0 (0.0)		1 (1.0)
Technical	10 (19.6)	1 (2.2)		2 (4.2)	9 (18.4)		11 (11.3)
Toxicity/morbidity	0 (0.0)	5 (10.9)		3 (6.3)	2 (4.1)		5 (5.2)
Other	2 (3.9)	1 (2.2)		2 (4.2)	1 (2.0)		3 (3.1)
Timing of primary outcome (months)	60 (24–60) (0.5–240)	60 (60–84) (3–240)	0.044	60 (60–120) (0.5–240)	60 (24–60) (1–240)	0.004	60 (36–60) (0.5–240)
Current status							
Open to recruitment	27 (52.9)	12 (26.1)	0.005	18 (37.5)	21 (42.9)	0.702	39 (40.2)
Closed to recruitment^d^	17 (33.3)	31 (67.4)		25 (52.1)	23 (46.9)		48 (49.5)
In set up	6 (11.8)	3 (6.5)		4 (8.3)	5 (10.2)		9 (9.3)
Unknown	1 (2.0)	0 (0.0)		1 (2.1)	0 (0.0)		1 (1.0)
Explicit patient involvement	3 (5.9)	3 (6.5)	0.896	5 (10.4)	1 (2.0)	0.087	6 (6.2)
Funding							
Government	11 (21.6)	13 (28.3)	0.479	17 (35.4)	7 (14.3)	0.003	24 (24.7)
Charity	8 (15.7)	11 (23.9)		11 (22.9)	8 (16.3)		19 (19.6)
Industry	6 (11.8)	3 (6.5)		0 (0.0)	9 (18.4)		9 (9.3)
Not stated	26 (51.0)	19 (41.3)		20 (41.7)	25 (51.0)		45 (46.4)

*IQR* interquartile range, *NST* neoadjuvant systemic therapy, *RT* radiotherapy.

*Involving more than one country/geographical are;

^a^Trials where recruitment is complete that have reported planned sample size and final actual recruitment numbers only (*n* = 44).

^b^2 surgical RCTs and one RT RCT have co-primaries—SMALL re-excision and LRR; ATNEC patient reported lymphoedema and survival and EUROPA – health-related quality of life and time to ipsilateral breast tumour recurrence.

^c^includes three studies of de-escalation of ‘axillary treatment’ (POSNOC, ATNEC, BOOG 2013-07) as surgery was standard of care for the control group at study initiation.

^d^includes trials that are complete *n* = 18, in follow up *n* = 23 or terminated *n* = 7.

^e^Kruskal-Wallis.

^f^Chi square test unless otherwise stated.

Compared with cohort studies, RCTs were more likely to be multicentre, international, government-funded studies with larger planned and actual recruitment numbers, and longer study durations. Cohort studies were significantly more likely to measure their primary outcome at an earlier timepoint, achieve their target accrual and receive industry funding than RCTs (Table 1).

Of the 97 studies, 51 (52.3%)^20,27,31,32,34,40,73,74,76,80,81,89,93,95,96,105^ and 46 (47.4%)^12,33,41,42,46,57,72,75,82,88,94,97,101–104^ evaluated de-escalation of surgery and radiotherapy respectively.

Compared with radiotherapy studies, surgical de-escalation trials were more likely to focus on reduction/omission of treatment to regional nodes (vs. breast) and treatment after NST. Studies de-escalating radiotherapy (RT), by contrast were more likely to involve international recruitment and be biomarker stratified (Table 1).

Details of trial design were available for 73/97 (75.3%) trials: (43/48 (89.6%) RCTs and 30/49 (61.2%) cohorts). Most RCTs (38/43, 88.4%) were non-inferiority trials with oncological primary endpoints (34/38, 89.5%). Reported non-inferiority margins (*n* = 27) ranged from 1.25% to 10% (median 3.6%) with no differences between locoregional recurrence (LRR) vs survival endpoints or surgery and radiotherapy trials (data not shown) (Supplementary Table 3). When reported, most cohort studies (29/30, 96.7%) stated basing their sample size calculation on a pre-defined acceptable threshold often for LRR (17/29, 58.6%) or a composite (6/29, 20.7%) endpoint including survival (e.g. disease-free or event-free survival). Reported acceptable LRR thresholds (14/17) were consistently <10% (median 4.5%, range 2% to >10%) whereas thresholds for composite survival-based outcomes were more variable (range >98.5% to >84%) with reductions of up to 10% considered acceptable in some studies (Supplementary Table 3). Only 21 studies^17,21,26,40,42,46,48,51,53,55,64,67,71,79,84,91,96^, however, reported how non-inferiority margins or (un)acceptable thresholds were selected. Over half (*n* = 11) reported basing estimates on the published literature or those used in previous/ongoing trials and over a quarter (*n* = 6) stated the decision had been made exclusively by study team. Few studies (*n* = 4) referred to engagement with the wider breast cancer community or explicitly stated that patient advocates had been involved in these decisions (*n* = 4). Statistical power was reported for 41/97 (42.3%) trials and was mostly 80% (31/41 75.6%) with only one quarter (10/41, 24.4%) reporting 90% power or higher.

**Fig. 2:**
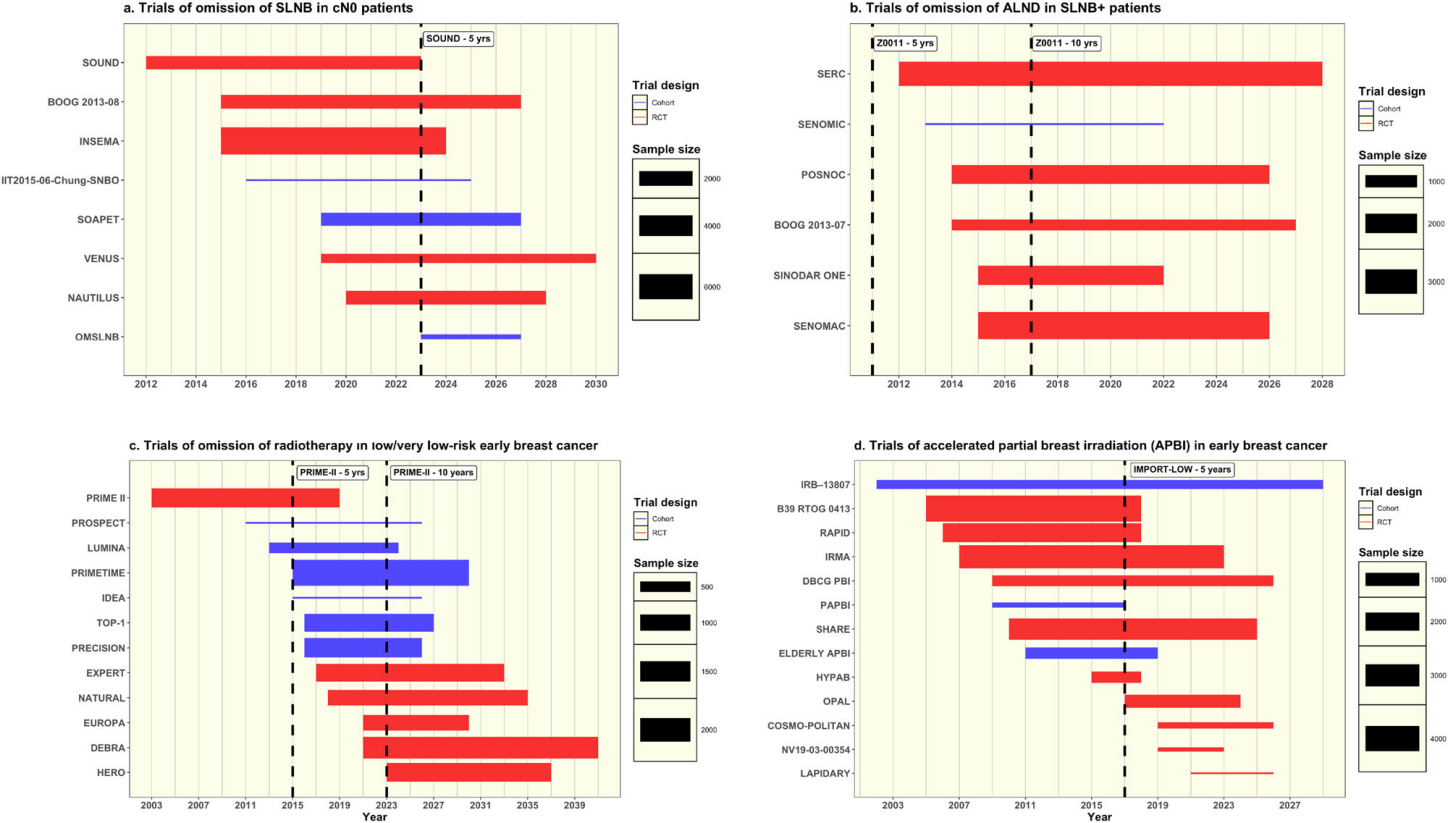
Concurrent locoregional treatment de-escalation studies including dates of publication of first pivotal study. **a** Trials of omission of SLNB in cN0 patients. **b** Trials of omission of ALND in SLNB+ patients. **c** Trials of omission of radiotherapy in low/very low risk early breast cancer. **d** Trials of partial breast irradiation (PBI) in early breast cancer.

Locoregional therapy de-escalation research questions could be broadly divided into two groups: i) evaluation of response-adjusted locoregional treatment in patients having NST and ii) omission/reduction of surgery or radiotherapy in patients with low- or intermediate-risk disease (Table 2, Supplementary Table 2). In both settings, multiple concurrent studies, often in different geographical locations, addressing identical or similar research questions in different or overlapping patient populations were identified (Fig. 2).

**Table 2:** Current landscape of locoregional de-escalation studies by treatment modality and research question

Surgery	Response adjusted treatment post NST		Primary surgery	
	Breast	Axilla	Breast	Axilla
**Omission of surgery**	**Omission of surgery patients with pCR on biopsy post NST** • EXCEPTIONAL RESPONDERS • OPTIMIST • ELPIS* • NOSTRA (F) • PetrovRIO	**Omission of axillary staging in cN0 PTS with complete response post NST** • BC-P29 • ASICS • ASLAN • EUBREAST-01	**Omission of surgery in small low risk unifocal cancers** • THERMAC (F) • COOL-IT (CA) • SMALL (VAE) • ICPS002/20 (Cool-tip) • RAFAELO (RFA) • TTUHSC IRB #L18-100 (CA) • ICE 3 (CA) • SCHBCC-N024 (CA) • FIRST (CA) • FROST (CA) • BR-003 (laser) • MINIVAB (VAE)(F)	**Omission of SLNB in cN0 patients** • SOUND• INSEMA • BOOG 2013-08 • NAUTILUS • VENUS • SOAPET • OMSLNB • IIT2015-06-Chung-SLNBO **Omission of SLNB in N1 patients** • ShandongCHI-13 (VAE)
**Reduction in extent of surgery**	**Omission of mastectomy in patients with inflammatory breast cancer who have complete response** • ConSIBreC	**Omission of ALND in patients either cN1 to cN0/ypN0 or pCR** • CTRI/2022/07/044461 • ATNEC • EUBREAST-2 INDAX^w^ • SrLNB • SLIBC • GANEA3 (F) • NeoaPET **Omission of SLNB in N1 patients** • MEDIPOL HOSP 1 **Omission of ALND IN ypN+ disease** • TAXIS • ALLIANCE A011202 (ypSLNB+) • ADARNAT (ypSLNB+) • EUBREAST-2 INDAX^w^ – cN+ TAD/SLNB • NEONOD2 (micros)	**De-escalation of mastectomy for multiple ipsilateral cancers** • ALLIANCE Z11102 • MIAMI^a^ (F)	**Omission of ALND in SLNB+ patients** • SERC • SENOMAC • SINODAR ONE • SENOMIC (micros) **Omission of axillary treatment (ALND/RT) in SLNB+ patients** • BOOG 2013-07 • POSNOC **TAS+RT vs ALND in cN+ patients** • TAXIS

Radiotherapy	Response adjusted treatment post NST		Primary surgery	
	Breast/chest wall	Axilla/regional nodes	Breast/chest wall	Axilla/regional nodes
**Omission of radiotherapy**	**Omission of breast RT in node negative early breast cancer achieving pCR post-NST** • DESCARTES (T1-2, N0) • ROSALIE • HER2noRT^a^ (HER-2 positive) **Omission of PMRT in patients having good response to NST** • NEMESIS		**Omission of BRT in low/very low-risk early breast cancer** • DEBRA* (ER+) • EUROPA (Luminal A, >70yrs) • EXPERT* (Luminal A, T1) • HERO (HER-2 +) • NATURAL • PRIME-II (ER+, >65yrs) • IDEA* (premenopausal women) • LUMINA*(Luminal A) • PRECISION* • PRIMETIME* • PROSPECT TOP-1 (>70yrs) **Omission of pmrt in intermediate risk patients (N1)** • SUPREMO • PORT-N1 • TAILOR RT* (low risk N1)	
**Reduction in target volumes**		**Omission of axillary RT in patients either cN1 to cN0/ypN0 or pCR**• ATNEC • EUBREAST-02-INDAX	**Partial breast irradiation in early breast cancer** • B39 RTOG 0413 • COSMOPOLITAN • DBCG PBI • HYPAB^a^ • IRMA • LAPIDARY • NC19-03-00345 • OPAL • RAPID • SHARE (postmenopausal) • ELDERLY APBI (low risk, >60yrs) • IRB-13807 (multiple modalities, inc IORT and brachytherapy) • PAPBI (F)(preoperative APBI) **Intraoperative radiotherapy (IORT)** • TARGIT-A • CTPR-0009 • IORT2 • UMIN000003578 • TARGIT C • TARGIT E (>70yrs T1) **Interstitial brachytherapy in early breast cancer** • GEC-ESTRO APBI • SiFEBI (MIB, 69-89yrs) • TRIUMPH-T **pCR after single dose of MRI guided pre-op PBRT in low-risk breast cancers** • ABLATIVE-2	**Omission of axillary/regional RT in SLNB+ patients** • OPTIMAL^a^* • T-REX (regional RT includes axilla, SCF ± IMC) • POSNOC • BOOG 2013-07 **Omission of regional nodal irradiation (RNI)** • KROG1701 (pN1 having BCS + WBI) • PORT-N1 (pN1 omission of RNI in BCS+WBI group) • TAILOR RT* (pN1 ER+ve HER-2 neg) **Omission of IMC RT IN early-stage intermediate risk (N1) patients** • IMNI PRECISION*

(F) Feasibility, Black—single arm cohort; Blue—RCT; Green—other non-randomised interventional design;

*BRT* breast radiotherapy, *CA* cryoablation, *ER* oestrogen receptor, *IMC* internal mammary chain, *MIB* multicatheter interstitial high dose rate brachytherapy, *micros* micrometastases, *NST* neoadjuvant systemic therapy, *pCR* pathological complete response, *PBRT* partial breast radiotherapy, *RT* radiotherapy, *SCF* supraclavicular fossa, *SLNB* sentinel lymph node biopsy, *VAE* vacuum-assisted excision, *WBI* whole breast irradiation

^*^Biomarker stratified;

^a^Terminated early;

Just over a quarter of trials (*n* = 26, 26.8%), predominantly early-phase and cohort studies (*n* = 18/26, 69.2%), evaluated de-escalation of surgery (*n* = 22)^15,20,32,54,71,80,87,89,98,100^ or radiotherapy (*n* = 6)^11,20,35,64,72,94^ in patients who responded well to NST (Table 2, Supplementary Table 2). This included one study de-escalating ‘axillary treatment’ (surgery or radiotherapy)^11^ and an RCT for cN1 patients that was withdrawn due to lack of funding^20^.

Five studies evaluated omission of breast surgery^66,68,85,87,89^ in patients with a complete response to NST. Of these, two^85,89^ were specifically early-phase feasibility studies while the remaining three aimed to determine oncological safety at a minimum of 3 years^66,68,87^. A further four omission of axillary surgery studies, aimed to assess the oncological safety of this approach following complete response to NST^67^.

Six studies (RCTs *n* = 2^11,32^, cohorts *n* = 4^71,83,98,100^) aimed to evaluate omission of axillary lymph node dissection (ALND) in patients converting from cN1 to cN0/ypN0 following NST. Cohort studies were either small single-centre trials^98,100^ or feasibility studies^71,83^, with two large RCTs^11,32^ investigating the impact of omitting ALND on five-year disease free survival (DFS) in a planned total of 4216 patients. Fewer studies (three RCTs^9,10,54^ and one cohort^84^) investigated omission of ALND in patients with ypN+ disease, and these aimed to assess oncological safety at a minimum of 5 years.

In many of the studies using response to NST as a biomarker for patient selection, definitions and inclusion criteria were not standardised. Definitions of ‘complete response’ ranged from clinical assessment alone, through to inclusion of imaging ± demonstration of a pathological complete response (pCR) in the breast. Studies omitting ALND in ypN+ disease varied in the burden of residual axillary disease permitted for study inclusion.

Three cohort studies^64,72,94^ aimed to investigate the oncological safety of omitting RT following breast conserving surgery (BCS) in cN0 patients achieving pCR, with one terminated prematurely due to recruitment issues^72^. One large RCT focused on omission of postmastectomy radiotherapy (PMRT) in patients with good treatment response^35^ and one RCT aimed to address omission of axillary radiotherapy in cN1 patients who had converted to ypN0 following treatment^11^.

The majority of studies in the portfolio (71/97, 73.2%) evaluated locoregional treatment de-escalation in patients having primary surgery, most commonly the omission of surgery or radiotherapy in patients with very low-risk disease.

In total, 12 concurrent, geographically diverse studies (3 RCTs^16,48,55^ and 9 cohorts^62,69,70,73,74,81,93,95,105^) commenced between 2014 and 2022 evaluating minimally-invasive alternatives to lumpectomy in patients with small, unifocal, node negative cancers. Most (*n* = 7/12, 58.3%) focused on cryoablation^16,69,70,73,74,95,105^, with the remainder studying a range of techniques including vacuum-assisted excision^48,81^, laser^62^ and radiofrequency ablation^93^. Many were early-phase and over half (7/12) included completeness of excision (a technical, feasibility outcome) post-procedure as a primary^55,62,69,70,74,81^ or co-primary^48^ endpoint.

Eight studies (5 RCTs^14,27,34,49,56^ and 3 cohorts^76,86,99^) aimed to recruit a total of 14,958 patients to assess the 5-year oncological safety of omitting SLNB. Most studies recruited clinically node negative patients aged 18 or over with T1-2 disease^14,27,34,56,99^^,^ and all but one study^76^ required confirmation of node negativity with one^14,27,34,49,56^ or more^86,99^ imaging modality. Only one study restricted recruitment to patients over 65^76^ and only two limited their inclusion criteria to smaller tumours^49,86^. Most studies required participants to undergo breast conserving surgery followed by radiotherapy and any additional recommended adjuvant treatments^14,27,34,56,76,99^ with only one study permitting mastectomy as a surgical treatment option^56^. Of the five RCTs, four involving 10,437 patients have completed recruitment^14,27,34^ and the earliest trial published results in 2023^49^ (Fig. 2a).

A total of 12 trials (6 RCTs^19,21,22,24,33,41^ and 6 cohort studies^75,79,91,92,103,106^) with a combined recruitment target of 13,145 patients aimed to evaluate the oncological safety of omitting breast radiotherapy following BCS in patients at very low risk of disease recurrence. Almost all (11/12, 91.7%) had a LRR primary endpoint assessed between 2.5 and 10 years (median 5 years). Studies recruited patients with ‘low risk’ T1N0 breast cancer but varied regarding inclusion criteria and how recurrence risk was assessed: several exclusively recruited older women^21,33,41,103^ and/or those with specific disease subtypes^19,21,22,24,41^. Several specifically recruited patients with luminal A disease^21,22,79^ determined either by assessment of ki67^79,91^ in combination with clinicopathological features (e.g. St Gallen Criteria) or using gene expression profiling with Prosigna/PAM50^22,106^. Other studies used Oncotype DX^19,75^ scores for risk stratification in similar patient populations. A single study used clinicopathological features and imaging with MRI to identify a low-risk group for omission for radiotherapy^92^. Most studies required patients to take adjuvant endocrine therapy for at least 5 years, but one study randomised participants to receive either partial breast radiotherapy or endocrine therapy^21^. Although the first radiotherapy omission trial opened in 2003 and has now published both 5-^107^ and 10-year^41^ outcomes, five large, multicentre RCTs addressing similar research questions^19,21,22,24,33^ and involving up to 5,989 patients are currently recruiting (Fig. 2c).

Reduction in target volume with external beam partial breast irradiation (PBI) in women with low-risk disease having BCS was evaluated in 13 studies (10 RCTs^12,17,18,25,28,30,36,37,42,46^ and 3 cohorts^65,78,88^) which opened between 2005 and 2019 with a combined recruitment target of 16,249 women (Fig. 2d). There were small but significant differences in inclusion/exclusion criteria across all studies. Almost all restricted recruitment by age, although the age criteria varied, with patients aged >40^42,78^, >50^30,36,37,46^, >55^25^, >60^18,88^ eligible for inclusion in different trials. Similarly, most trials restricted inclusion by tumour size, most commonly <3 cm^12,28,30,37,42,88^, but some studies only recruited patients with T1 disease^25,46^ or tumours <2.5 cm^36,78^. Consistent with identifying a ‘low risk’ population, almost all studies only included patients with node negative disease, but two also recruited N1 patients^12,28^. There was significant variability in the tumour grade and biological subtypes of cancer included in different studies and in the surgical resection margin required for study entry. This was most commonly >2 mm^28,30,46,78^ but ranged from ‘no tumour on ink’^37^ to >5mm^25^ in different trials.

Of the 13 studies, most have completed; two RCTs^25,46^ were terminated early due to recruitment issues and two remain open^17,30^. Most (*n* = 8/13, 61.5%) focused on oncological safety with LRR endpoints at between 5 and 20 years^12,28,42,46,65,78,88^ but radiotherapy-related toxicity^18,36,37^, cosmesis^25,36^ or quality of life^17^ were also evaluated at variable timepoints.

Nine trials (2 RCTs and 7 cohorts) opened between 2000 and 2015 to evaluate PBI using intraoperative radiotherapy^53,63,77,82,101,102^ or brachytherapy^23,97,104^. These have all completed recruitment.

A total of 13 trials (12 RCTs and 1 cohort study) de-escalating either surgery^13,40,43,44,47,54,96^ or radiotherapy^13,26,29,50–52^ in patients with intermediate-risk pN1 disease were identified.

Five large European RCTs^13,40,43,44,47^ aimed to assess the oncological safety of omitting ALND in SLNB+ patients in 11,930 patients (Fig. 2b) including two studies^13,40^ evaluating the omission of axillary treatment (either ALND or nodal radiotherapy). Most trials recruited patients aged 18 or above^13,40,43,44^ with clinically node negative T1-2 breast cancer^13,40,44,47^. Only one study included T3 disease and a further study restricted recruitment to patients aged between 40 and 75 years^47^. Three studies required US assessment of the axilla as part of the entry criteria^13,43,47^. Most permitted either BCS or mastectomy, but one study included only patients undergoing mastectomy^13^. Inclusion criteria regarding the degree of sentinel node involvement were also variable. Some studies mandated patients to have one or two sentinel node macro-metastases^40,43^ whereas others permitted inclusion of patients with micro-metastases^13^ or isolated tumour cells in their sentinel nodes^44^.

Of the five trials, three did not meet their recruitment targets, with two closed due to recruitment issues^13,47^. One of these later re-opened as single arm mastectomy cohort^108^. Only one is currently recruiting^44^.

Only one large RCT recruiting cN1 patients having primary surgery was identified and is currently open with participants randomised to either targeted axillary surgery and radiotherapy or ALND^54^.

Two RCTs aimed to assess the impact of omitting axillary and/or regional radiotherapy on 5-year survival in patients with limited pN1(sn) disease^38,51^, one of which was terminated prematurely due to poor recruitment^38^.

Four further RCTs aimed to assess the oncological safety of omitting regional nodal irradiation (RNI)^29,39,52^ or internal mammary chain (IMC) radiotherapy^26^ in intermediate-risk N1 patients. Two studies^26,52^ used biomarker stratification to assess risk and two included omission of PMRT^39,52^ in mastectomy patients randomised to omission of RNI. Omission of PMRT alone in intermediate-risk patients was also assessed in a further RCT^50^. One study closed due to poor recruitment^29^, one is in follow-up^50^ and the remainder are currently recruiting.

## Discussion

This systematic review provides a comprehensive overview of the current landscape of locoregional therapy de-escalation trials in early breast cancer, which is focused on two broad areas: i) response-adjusted locoregional treatment in patients having NST and ii) omission/reduction of locoregional treatment in patients with low to intermediate-risk disease.

There is, however, significant duplication of effort across the portfolio, with multiple studies including several concurrent large-scale RCTs aiming to address identical or similar research questions. This was particularly evident in low-risk patients, with nine SLNB omission studies, 12 radiotherapy omission studies and 13 PBI studies with planned accrual of 12,919, 19,134 and 16,249 patients respectively identified. There were also five concurrent European RCTs evaluating omission of ALND in the SLNB+ population.

Challenges were highlighted with current de-escalation study design. It is increasingly stated that tumour biology rather than anatomy is key to determining outcomes but currently less than 10% of trials in the portfolio use biomarker stratification to inform participant selection and/or treatment. Although response to NST is a dynamic biomarker which is being utilised in a proportion of trials, the ongoing use of traditional methods of risk-stratification such as pathological stage in studies de-escalating locoregional treatment in patients with low- and intermediate-risk disease is a concern. Furthermore, future studies should consider not only the use of prognostic biomarkers to determine level of risk, but also whether predictive biomarkers can identify patients who will not benefit from specific treatments, enabling de-escalation based on biology as well as risk of recurrence.

As expected, most studies have an oncological primary endpoint and are designed to assess either non-inferiority or ‘acceptable’ levels of oncological safety at a minimum of 5-years. This is problematic as most patients have low risk disease with few recurrences. This results in large, long, and expensive studies with few events that may be underpowered, often struggle to recruit and risk practice changing in the interim without evidence, which may result in patient harm.

Justification for multiple concurrent large-scale RCTs addressing similar or identical research questions is challenging but may relate to lack of engagement of patients and the wider breast cancer community highlighted by this review. Patients are extremely supportive of research to improve personalisation of care^3^, but less than 10% of trials explicitly stated involving patient advocates in the study design. Similarly, few trials engaged with clinicians outside the immediate study team. Failure to engage the broader breast cancer community in both the choice of research question and study design can jeopardise future implementation. Lack of engagement and ownership increases the risk that trials will be criticised and reduces the likelihood that the findings will be implemented. Results of the ACOSOG Z0011 trial^109,110^, for example, were not accepted in Europe due to concerns about inclusion criteria, event rate and lack of radiotherapy quality assurance^111^. Five large-scale RCTs^13,40,43,44,108^ were subsequently developed to validate the results, delaying patient benefit and creating an opportunity cost to the breast cancer community through inefficient use of research funding.

This review highlights a lack of a co-ordinated global research across the entire locoregional de-escalation portfolio but similar issues also exist in the systemic therapy context^112^. There is therefore a need for the international breast cancer community to work together with patients to develop efficient, credible, well-designed studies that can and will be implemented globally.

There are limitations to this review which require consideration. Firstly, it has relied on publicly available data sources for information on included trials. While many trials have published either protocols or preliminary results, for many ongoing trials, only the trial registry record was available. This information is limited and may not include details such as patient involvement in study design or information on sample size calculations such that some of these parameters were underestimated. It is also possible that the search strategy may have failed to identify all potentially relevant trials. A combination of medical database and trials registry search was chosen to minimise this risk. Trial registration is mandatory, so it is unlikely that any potentially practice changing studies were missed. This review is therefore likely to present a comprehensive picture of the current locoregional de-escalation landscape and the areas where improvement is required.

There is an urgent need to address the current slow, inefficient and siloed approach to the design and delivery of locoregional de-escalation studies to optimise the value of future research and prevent research waste. This will involve international collaboration and engagement of the global breast cancer community, not only to agree key research priorities but also to ensure that future trials are well-designed and credible so that the results are implemented into practice globally. Patients want research to demonstrate oncological safety^3^, but recurrence in this patient population is now rare, so traditional RCTs powered to demonstrate non-inferiority for oncological endpoints are increasingly numerically unfeasible. Large sample sizes could be addressed by co-ordinated international recruitment or harmonisation of trial protocols particularly inclusion criteria and endpoint selection with agreed core outcome sets for locoregional de-escalation studies to facilitate data pooling and pre-planned individual patient level meta-analysis, which are currently hampered by the heterogeneity described above. This would require global collaboration and consensus, but should be achievable. While both strategies are undoubtedly part of the future solution, however, more innovative and efficient study designs such as trials within cohorts^113^ or platform studies^114^, facilitating exploration of multiple research hypotheses simultaneously, will be essential to generate timely results to inform practice. A platform approach, for example, could evaluate multiple potential approaches to risk stratification for radiotherapy omission, increasing efficiency and reducing costs by obviating the need for multiple separate trials. This, together with increased use of biomarkers to appropriately stratify participant risk and promote greater personalisation of care will create an efficient, responsive de-escalation portfolio. Finally, and most importantly, engagement with patient partners will be essential in the design and development of all future treatment de-escalation studies. Only then can we ensure such studies answer clinically relevant questions for the breast cancer community and are feasible, acceptable and deliverable.

## Methods

This systematic review was prospectively registered on the PROSPERO International Register of Systematic Reviews (CRD42023487777) and is reported according to PRISMA guidelines^115^.

A comprehensive search of online databases (OVID, Medline, Embase and the Cochrane Library) and English language trial registries (clinicaltrials.gov, ISRCTN, Dutch trials registry, and European clinical trials registry platform) was undertaken on the 16/01/2024 to identify ongoing or recently completed trials evaluating the de-escalation of locoregional therapy in patients with early breast cancer. The search strategy was developed and iteratively refined using terms for ‘breast cancer’, ‘surgery’, ‘radiotherapy’ and ‘de-escalation’ and adapted for use in the trial registries. Details of the searches performed are summarised in Supplementary Table 4. The search was limited to studies published on or after 01/01/2019 to focus on the contemporary de-escalation portfolio.

All phase 2 and 3 randomised controlled trials (RCTs) and non-randomised interventional cohort studies evaluating de-escalation of locoregional treatment in adult patients >18 years with early invasive breast cancer that were in set-up, ongoing, had recently terminated or been published in English between 01/01/2019 and 31/12/2023 were eligible for inclusion. Excluded were observational (non-interventional) studies, reviews, letters and editorials. Studies investigating de-escalation of systemic breast cancer therapy were also excluded, but those evaluating de-escalation of locoregional treatments following neoadjuvant systemic therapy (NST) were eligible for inclusion.

Citations were uploaded to Zotero® software and duplicates removed. Remaining studies were screened for inclusion using pre-specified inclusion criteria by one reviewer (ADMcC). All potentially relevant studies and those in which eligibility was unclear were discussed by the study team at regular meetings and inclusion of all studies agreed by consensus.

For the purposes of this review, early breast cancer was defined as Stage 1–3 disease treated with curative intent. Locoregional treatment was defined as surgery or radiotherapy to the breast and/or regional nodal areas.

De-escalation was defined as either i) a reduction in the extent or ii) complete omission of surgery or radiotherapy. For radiotherapy, this included any reduction in target volume (e.g. partial breast irradiation) but not method of delivery, dose or fractionation.

Biomarker stratification was defined as selection of patients for treatment based on any biological, molecular or genetic information over and above that used for routine breast cancer diagnostics (i.e. ER/PR/HER2). This included Ki67, gene expression profiling and other genomic tests.

A data extraction tool was developed, iteratively refined and piloted in Microsoft Excel® by the study team. Data extracted included study name, registry number, number of sites, geographical location; design; modality of locoregional treatment de-escalated, site at which treatment was reduced/omitted (breast or nodal areas), use of biomarker stratification, primary outcome, source of funding and current status. Study start/end dates; planned/actual duration of recruitment and planned/actual recruitment numbers were also extracted. Finally, specific details related to the research question, study design, hypothesis, sample size calculation and patient and public involvement were extracted verbatim. All publicly available data sources including study protocols, published conference proceedings/abstracts and registry records were used to optimise data completeness. Data extraction was performed by one reviewer (ADMcC); the study team met regularly throughout the data extraction process to review the data and discuss and resolve uncertainties. A proportion (approximately 10%) of studies were independently reviewed by senior members of the team (SP, SAMcI) to ensure consistency and methodological rigor.

Simple descriptive statistics were used to summarise the results. Categorical data were summarised by counts and percentages and continuous data by median, interquartile range (IQR) and range. Characteristics of included studies were compared by i) study design (RCT vs cohort) and ii) treatment modality de-escalated (surgery vs. radiotherapy) using chi-squared and Kruskal-Wallis tests for categorical and continuous variables respectively. Extracted verbatim data regarding study design, research question and hypothesis testing were explored using content analysis^116^ and a narrative synthesis was performed. No risk of bias assessment or pooling of the data was undertaken as the aim of the review was to describe the landscape of locoregional breast cancer de-escalation studies.

## Supplementary information

Supplementary material

## Data Availability

All data from this review are presented in the manuscript, supplementary material and reference list. No further data are available.
