# Supplementary material for: Mapping the current landscape of locoregional therapy de-escalation trials in early breast cancer: a systematic review
Source: NPJ Breast Cancer. 2025 Mar 30;11:32. doi: 10.1038/s41523-025-00744-9 (PMC11955517; doi:10.1038/s41523-025-00744-9)
Supplement: Supplementary file 1 — Supplementary material [file 41523_2025_744_MOESM1_ESM.docx]

**Mapping the current landscape of locoregional therapy de-escalation trials in early breast cancer: a systematic review**

**Supplementary material**

**Table of contents**

Supplementary Table 1: Studies excluded after review by study team Page 2

Supplementary Table 2: Included de-escalation studies by research question Page 3

Supplementary Table 3: Study designs and hypothesis testing Page 13

Supplementary Table 4: Search strategies Page 16

**Supplementary Table 1: Studies excluded after review by study team**

| **Reason for exclusion** | **Study Name** | **Registry number** |
| --- | --- | --- |
| **Pre-invasive disease (DCIS) (n=15)** | AAAQ7853 | NCT03216421 |
|  | COMET | NCT02926911 |
|  | DCIS CRYO | NCT05218044 |
|  | DUCHESS | NCT02766881 |
|  | ECOG-ACRIN E4112 | NCT02352883 |
|  | HORNEO 01 | NCT04666961 |
|  | IORT-WRT-DCIS | NCT02389699 |
|  | LORD | NCT02492607 |
|  | LORETTA | UMIN000028298 |
|  | LORIS | ISRCTN27544579 |
|  | PRISCILLA | NCT05663294 |
|  | RECAST-DCIS | NCT06075953 |
|  | ROMANCE | NCT03878342 |
|  | SentiNOT | ISRCTN18430240 |
|  | TBCC-ARO DCIS | NCT04046159 |
| **Not locoregional treatment de-escalation (n=10)** | N/A | IRCT20180919041070N2 |
|  | OPAR | NCT02637024 |
|  | KROG 0806 | NCT04803266 |
|  | B-51 RTOG 1304 | NCT01872975 |
|  | N/A | NCT01452672 |
|  | EndoNET | ISRCTN11896599 |
|  | POTENTIAL | NCT04320979 |
|  | OFF-MAP | NCT03975179 |
|  | MAGTOtal | ISRCTN11914537 |
|  | TARGIT-B | NCT01792726 |
| **Non-interventional study**  **(n=5)** | N/A | NCT06039956 |
|  | RAPCHEM | NCT01279304 |
|  | NEO SENTITURK | NCT04250129 |
|  | 939530lp | NCT02860104 (?) |
|  | S2020-09 | NCT04626986 (?) |
| **Not primary breast cancer (n=2)** | RTOG 1014 | NCT01082211 |
|  | VAPEX | NCT03868475 |

**Supplementary Table 2: Included de-escalation studies by research question**

| **RESPONSE-ADJUSTED LOCOREGIONAL TREATMENTS IN PATIENTS FOLLOWING NEOADJUVANT SYSTEMIC THERAPY (NST)** | | | | | | | | | | | |
| --- | --- | --- | --- | --- | --- | --- | --- | --- | --- | --- | --- |
| **OMISSION OF SURGERY PATIENTS WITH pCR ON BIOPSY POST NST** | | | | | | | | | | | |
| **Name** | **Trial registry ID** | **Primary location** | **Multi-centre** | **Design** | **Research question** | **Biomarker stratified** | **Planned sample size** | **Final sample size** | **Primary outcome** | **Start year** | **Status** |
| EXCEPTIONAL RESPONDERS | NCT02945579 | USA | Yes | Single-arm prospective cohort | This clinical trial studies eliminating surgery and how well radiation therapy after systemic therapy works in treating patients with HER2 positive or TNBC when image-guided biopsy shows no residual cancer. Patients then receive standard breast radiotherapy. | No | 120 | NA | Ipsilateral breast tumour recurrence-free survival | 2017 | Recruiting |
| OPTIMIST | NCT05505357 | South Korea | Yes | Single-arm prospective cohort | Are breast cancer patients who are predicted to have a pCR on MRI and VAB after NST, and are omitted breast surgery likely to have a non-inferior 5-year DFS compared to those who had received breast surgery? | No | 533 | NA | Disease free survival | 2022 | Recruiting |
| ELPIS | NCT04578106 | Spain | No | Single-arm prospective cohort | To estimate the loco-regional invasive DFS at 3-year of patients who achieve a complete response based on imaging (i.e. MRI) and a stereotactic-guided VAB biopsy and omit loco-regional surgery. | Yes | 17 | NA | Loco-regional invasive disease-free survival | 2020 | Recruiting |
| NOSTRA | ISRCTN17170034 | UK | Yes | Single-arm prospective cohort | Is it safe to omit surgery after planned NST plus dual-targeted anti-HER2 treatment and can patients with residual cancer can be identified by histological examination of multiple ultrasound-guided tumour bed core biopsies following dual-targeted neoadjuvant treatment for HER2-positive, ER-negative early primary breast cancer and is there is concordance between local and central pathology reporting? | No | 150 | NA | Feasibility of future larger clinical trial | 2019 | Recruiting |
| PetrovRIO | NCT04293796 | Russia | No | Two-arm prospective cohort | To study the viability (including false positive/negative biopsy rates post NST) of using US guided VAB as an alternative to surgical excision in unifocal stage IIA-IIIA HER2 positive or triple negative breast cancer after a good clinical response to NST | No | 60 | NA | False negative rate | 2019 | Recruiting |
| **OMISSION OF MASTECTOMY IN PATIENTS WITH INFLAMMATORY BREAST CANCER WHO HAVE COMPLETE RESPONSE** | | | | | | | | | | | |
| **Name** | **Trial registry ID** | **Primary location** | **Multi-centre** | **Design** | **Research question** | **Biomarker stratified** | **Planned sample size** | **Final sample size** | **Primary outcome** | **Start year** | **Status** |
| ConSIBreC | NCT06131632 | Italy | NS | RCT | To investigate the possible use of the BCS instead of mastectomy in patients with inflammatory breast cancer that achieve clinical complete response after NST. | No | 300 | NA | Local recurrence rate | 2024 | Not yet recruiting |
| **OMISSION OF AXILLARY STAGING IN cN0 PTS WITH COMPLETE RESPONSE POST NST** | | | | | | | | | | | |
| **Name** | **Trial registry ID** | **Primary location** | **Multi-centre** | **Design** | **Research question** | **Biomarker stratified** | **Planned sample size** | **Final sample size** | **Primary outcome** | **Start year** | **Status** |
| ASICS | NCT04225858 | Netherlands | NS | Single-arm prospective cohort | To evaluate whether SLNB can safely be omitted in breast cancer patients with HER2+ or TNBC tumours who achieve a radiological complete response on MRI after neoadjuvant systemic therapy. | No | 340 | NA | Axillary recurrence | 2020 | Not yet recruiting |
| BC-P29 | NCT05314114 | China | No | Single-arm prospective cohort | To test the hypothesis that selective omission of axillary surgery in distinct responders after NST will not deteriorate survival. In the planned trial, axillary surgery will be completely eliminated for initially cN0 TNBC and HER2-positive breast cancer patients who achieve pCR in breast after NST determined by lumpectomy. | No | 136 | NA | Ipsilateral axillary recurrence-free survival | 2020 | Recruiting |
| EUBREAST-01 | NCT04101851 | Germany | Yes | Single-arm prospective cohort | To prove the oncological safety of omission of axillary SLNB after pCR in the breast in response to NST for TNBC and HER2-positive disease in initially clinical node-negative (cN0) patients. | No | 350 | NA | Axillary recurrence free survival | 2021 | Recruiting |
| ASLAN | NCT04993625 | South Korea | Yes | Single-arm prospective cohort | To evaluate 5 year RFS when SLNB is omitted after NST in triple negative or HER-2 positive breast cancer patients when physical examination and imaging show complete remission. | No | 178 | NA | Recurrence free survival | 2021 | Recruiting |
| **OMISSION OF AXILLARY STAGING IN cN0 PTS WITH COMPLETE RESPONSE POST NST** | | | | | | | | | | | |
| **Name** | **Trial registry ID** | **Primary location** | **Multi-centre** | **Design** | **Research question** | **Biomarker stratified** | **Planned sample size** | **Final sample size** | **Primary outcome** | **Start year** | **Status** |
| N/A | CTRI/2022/07/044461 | India | Ns | RCT | To show the non-inferiority of DFS between axillary conservative surgery (defined further as low axillary sampling or equivalent SLNB procedure) as compared to complete ALND in patients who are rendered cN0 after NST | No | 2316 | NA | Disease free survival | 2022 | Not yet recruiting |
| ATNEC | ISRCTN36585784 | UK | Yes | RCT | Will omitting further axillary treatment (ALND and ART) for patients with early-stage breast cancer and cN+ on needle biopsy, who after NST have no residual cancer in the lymph nodes on SLNB, be non-inferior to axillary treatment? | No | 1900 | NA | Disease free survival; patient reported lymphoedema | 2020 | Recruiting |
| SLIBC | NCT06130241 | Egypt | No | Single-arm prospective cohort | To determine the accuracy and safety of SLNB after NST in women who were initially axillary node positive but convert to negative after NST. | No | 30 | NA | Local recurrence | 2023 | Not yet recruiting |
| GANEA3 | NCT03630913 | France | Yes | Single-arm prospective cohort | To evaluate the interest of identifying, before NST, the initial involved lymph node to improve the prediction of axillary status after NST | No | 385 | NA | False negative rate | 2019 | Recruiting |
| SrLNB | NCT05939830 | China | No | Single-arm prospective cohort | To determine if ALND can be safely omitted in breast cancer patients with axillary pCR after NST | No | 92 | NA | invasive Disease-Free Survival | 2023 | Recruiting |
| NeoaPET | NCT05914402 | China | No | Single-arm prospective cohort | To evaluate the feasibility and safety of axillary surgery de-escalation for the cN+ patients who are predicted to achieve A-pCR using multiple pathological indicators and imaging examinations (before and after 1-2 cycles NST. | No | 100 | NA | Node recurrence rate | 2023 | Recruiting |
| **OMISSION OF SLNB IN N1 PATIENTS** | | | | | | | | | | | |
| **Name** | **Trial registry ID** | **Primary location** | **Multi-centre** | **Design** | **Research question** | **Biomarker stratified** | **Planned sample size** | **Final sample size** | **Primary outcome** | **Start year** | **Status** |
| MEDIPOL HOSP 1 | NCT06096545 | Turkey | Yes | 2-arm prospective cohort | Can axillary biopsy replace SLNB in the axillary assessment of women who have underwent NST? | No | 50 | NA | Prediction of axillary status | 2023 | Not yet recruiting |
| **OMISSION OF ALND IN ypN+ DISEASE** | | | | | | | | | | | |
| **Name** | **Trial registry ID** | **Primary location** | **Multi-centre** | **Design** | **Research question** | **Biomarker stratified** | **Planned sample size** | **Final sample size** | **Primary outcome** | **Start year** | **Status** |
| ALLIANCE A011202 | NCT01901094 | USA | Yes | RCT | To evaluate whether radiation to the undissected axilla and regional lymph nodes is not inferior to ALND with radiation to the regional lymph nodes but not to the dissected axilla in terms of invasive breast cancer recurrence-free interval in patients with positive ypSLNB+ after completion of NST. | No | 2918 | 2012 | Invasive breast cancer recurrence free interval | 2014 | Follow up |
| ADARNAT | NCT04889924 | Spain | YES | RCT | To evaluate whether ART presents a lower risk of lymphedema with respect to ALND and a non-inferior survival/disease recurrence outcome in patients with breast cancer who, after NST present the ypSLNB+. | No | 1660 | NA | Disease free survival | 2021 | Recruiting |
| EUBREAST-2 INDAX | NCT04281355 | Sweden | NA | RCT | To implement de-escalated staging and evaluate which regional treatment, individually adapted to the response after NST, is oncologically safe but least harmful. | No | 2946 | 0 | Invasive disease free survival | 2021 | Terminated |
| TAXIS | NCT03513614 | Switzerland | Yes | RCT | Is TAS and axillary RT non-inferior to ALND in terms of DFS of N+ breast cancer patients at high risk of recurrence in the era of effective systemic therapy and extended RNI? | No | 1500 | NA | Disease free survival | 2018 | Recruiting |
| NEONOD2 | NCT04019678 | Italy | No | 2-arm prospective cohort | To verify whether the omission of axillary lymph node intervention in patients with SLN ypN1mi after NST does not lead to a significant deterioration in survival or in the risk of regional or distant recurrence, compared to patients with SLN ypN0 after NST, where the omission of axillary treatment is currently the standard treatment. | No | 850 | NA | Disease free survival | 2019 | Recruiting |
| **OMISSION OF BREAST RT IN NODE NEGATIVE EARLY BREAST CANCER ACHIEVING pCR POST-NST** | | | | | | | | | | | |
| **Name** | **Trial registry ID** | **Primary location** | **Multi-centre** | **Design** | **Research question** | **Biomarker stratified** | **Planned sample size** | **Final sample size** | **Primary outcome** | **Start year** | **Status** |
| Her2noRT | NCT03460067 | USA | No | 3-arm prospective cohort | To describe the rate of local control in patients with HER2 positive early stage breast cancer with a complete response to chemotherapy and lumpectomy alone. | No | 78 | 12 | Local control rate | 2018 | Terminated |
| DESCARTES | NCT05416164 | Netherlands | Yes | Single-arm prospective cohort | Can radiotherapy be safely omitted in breast cancer patients with T1-2N0 tumours who achieve a pCR after neoadjuvant systemic therapy and BCS? | No | 595 | NA | Local recurrence | 2022 | Not yet recruiting |
| ROSALIE | NCT05866458 | Canada | Yes | Single-arm prospective cohort | To study the effect of radiation omission in patients with cN0 breast cancer undergoing lumpectomy and with a pCR after NST with regards to tumour recurrence. | No | 352 | NA | Ipsilateral breast tumour recurrence | 2023 | Not yet recruiting |
| **OMISSION OF PMRT IN PATIENTS HAVING GOOD RESPONSE TO NST** | | | | | | | | | | | |
| **Name** | **Trial registry ID** | **Primary location** | **Multi-centre** | **Design** | **Research question** | **Biomarker stratified** | **Planned sample size** | **Final sample size** | **Primary outcome** | **Start year** | **Status** |
| NEMESIS | NCT05993559 | South Korea | Yes | RCT | To confirm that in patients with breast cancer who have undergone mastectomy after prior NST, omitting RT is non-inferior to PMRT in terms of 5-year DFS in patients with pCR compared to patients treated with PMRT. | No | 1314 | NA | Invasive disease free survival | 2023 | Not yet recruiting |
| **DE-ESCALATION OF LOCOREGIONAL TREATMENTS IN PATIENTS WITH LOW/INTERMEDIATE-RISK DISEASE** | | | | | | | | | | | |
| **OMISSION OF SURGERY IN SMALL LOW RISK UNIFOCAL CANCERS** | | | | | | | | | | | |
| **Name** | **Trial registry ID** | **Primary location** | **Multi-centre** | **Design** | **Research question** | **Biomarker stratified** | **Planned sample size** | **Final sample size** | **Primary outcome** | **Start year** | **Status** |
| SMALL | ISRCTN12240119 | UK | Yes | RCT | To determine whether the extent of surgical treatment can be reduced in the context of standard adjuvant radiotherapy and endocrine therapy by performing minimally invasive VAE of small screen detected breast cancers instead of BCS. | No | 800 | NA | Re-excision rates and local recurrence rate | 2019 | Recruiting |
| THERMAC | NL9205 | Netherlands | No | RCT | The aim of this phase 2 screening trial is to determine the efficacy rate of radiofrequency, microwave and cryoablation with the intention to select one treatment for further testing in a phase 3 trial. | No | 63 | NA | Absence of residual disease | 2021 | Recruiting |
| COOL-IT | NCT05505643 | USA | No | RCT | To review the safety of the cryoablation procedure initially, followed by comparing cryoablation to lumpectomy in order to see if the cryoablation results in better disease control, complication rates, and quality of life. | No | 256 | NA | Ipsilateral breast cancer recurrence | 2024 | Not yet recruiting |
| FROST | NCT01992250 | USA | Yes | Single-arm prospective cohort | The hypothesis is that cryoablation will complete ablation and destroy the tumour in a selected population of women who may otherwise be adequately treated with surgery. | No | 200 | NR | Absence of residual disease | 2013 | Follow up |
| FIRST | NCT05398497 | Brazil | No | Single-arm prospective cohort | This study assesses whether cryoablation can be used as an alternative to surgery in cases of early invasive breast carcinoma. | No | 32 | NA | Absence of residual disease | 2022 | Recruiting |
| ICE 3 | NCT02200705 | USA | Yes | Single-arm prospective cohort | To evaluate the efficacy of cryoablation without lumpectomy and its impact on local and distant recurrence of early stage breast cancer. | No | 150 | 194 | In breast tumour recurrence | 2014 | Follow up |
| BR-003 | NCT03463954 | USA | Yes | Single-arm prospective cohort | To evaluate Novilase for the focal destruction of malignant tumours of the breast that are less than or equal to 15 mm against a performance goal for the standard of care | No | 122 | NA | Absence of residual disease | 2023 | Recruiting |
| MINIVAB | NCT04107636 | Netherlands | Yes | Single-arm prospective cohort | To assess whether it is feasible to remove small breast cancers <15mm completely using VAB under US guidance. | No | 170 | NA | Absence of residual disease | 2021 | Recruiting |
| ICPS002/20 | NCT04389216 | Spain | Yes | Single-arm prospective cohort | To validate the efficacy and safety of the cool-tip ablation method for breast tumours smaller than 2 cm in women aged 65 years and older. | No | 30 | NA | Absence of residual disease | 2020 | Recruiting |
| RAFAELO | UMIN000008675 | Japan | Yes | Single-arm prospective cohort | Evaluate the efficacy of RFA for early breast cancer to standardize it as local therapy in women with early invasive unifocal breast cancer (<1.5cm) | No | 372 | 372 | Ipsilateral breast tumour recurrence | 2013 | Follow up |
| SCHBCC-N024 | NCT04334785 | China | No | Single-arm prospective cohort | The long-term effectiveness and safety of cryo-ablation in early invasive breast cancer is still unknown. Therefore, this prospective study is designed to evaluate the effectiveness and safety of cryo-ablation in early invasive breast cancer. | No | 186 | NA | Loco-regional free survival | 2020 | Recruiting |
| TTUHSC IRB #L18-100 | N/A | USA | No | Single-arm prospective cohort | To study the medium to long term outcomes of women 50 years and older who underwent cryoablation for small (<15mm) unifocal invasive ductal carcinomas receptor profiled genetically and followed up by way of US and MRI. | No | 32 | 32 | Local recurrence | 2017 | Complete |
| **DE-ESCALATION OF MASTECTOMY FOR MULTIPLE IPSILATERAL CANCERS** | | | | | | | | | | | |
| **Name** | **Trial registry ID** | **Primary location** | **Multi-centre** | **Design** | **Research question** | **Biomarker stratified** | **Planned sample size** | **Final sample size** | **Primary outcome** | **Start year** | **Status** |
| ALLIANCE Z11102 | NCT01556243 | USA | Yes | Single-arm prospective cohort | To evaluate whether the LR rate with BCS followed by whole breast radiation with radiation boost to lumpectomy sites at 5 years is <8%. | No | 200 | 270 | Local recurrence | 2012 | Complete |
| MIAMI | ISRCTN17987569 | UK | Yes | RCT | Can patients with multiple breast cancers in the same breast avoid mastectomy by having multiple lumpectomies to achieve equivalent rates of local breast cancer recurrence? | No | 50 | 8 | Feasibility of future larger clinical trial | 2018 | Terminated |
| **OMISSION OF SLNB IN cN0 PATIENTS** | | | | | | | | | | | |
| **Name** | **Trial registry ID** | **Primary location** | **Multi-centre** | **Design** | **Research question** | **Biomarker stratified** | **Planned sample size** | **Final sample size** | **Primary outcome** | **Start year** | **Status** |
| SOUND | NCT02167490 | Italy | Yes | RCT | To determine whether the omission of axillary surgery is noninferior to SLNB in patients with small BC and a negative result on preoperative axillary lymph node US. | No | 1560 | 1463 | Distant disease free survival | 2012 | Complete |
| BOOG 2013-08 | NCT02271828 | Netherlands | Yes | RCT | To determine whether omitting the SLNB is not inferior to the current axillary staging regimen in cN0 breast cancer patients undergoing BCS, in terms of RR rate. | No | 1730 | 1735 | Regional recurrence rate | 2015 | Follow up |
| INSEMA | NCT02466737 | Germany | Yes | RCT | To show that early-stage breast cancer patients with reduced extent of axillary surgery are not inferior regarding DFS compared with those undergoing SLNB. | No | 7095 | 5505 | Invasive disease free survival | 2015 | Follow up |
| NAUTILUS | NCT04303715 | South Korea | Yes | RCT | To establish the minimally invasive treatment of breast cancer by re-examining the necessity of SLNB among patients with invasive breast cancer who have tumours under 5cm, are cN0, and are having BCS | No | 1734 | 1734 | Invasive disease free survival | 2020 | Follow up |
| VENUS | NCT05315154 | Brazil | Yes | RCT | Is omission of SLNB non-inferior to SLNB in a population of Latin American women with early breast cancer (tumour <5cm) and node negative after clinical and ultrasound exam with regards to five year DFS | No | 800 | NA | Disease free survival | 2019 | Recruiting |
| IIT2015-06-Chung-SNBO | NCT02564848 | USA | No | Single-arm prospective cohort | To determine whether omission of SLNB in patients who meet eligibility criteria results in an acceptable regional recurrence rate over a 6-year period. | No | 200 | NA | Regional recurrence | 2016 | Recruiting |
| OMSLNB | NCT05935150 | China | No | Single-arm prospective cohort | To study the omission of SLNB in patients with cN0 early breast cancer (tumour<3cm) whilst maintaining survival, decrease the oedema of upper arm, and finally improve the quality of life of the patients. | No | 311 | NA | Invasive disease free survival | 2023 | Recruiting |
| SOAPET | NCT04072653 | China | No | Single-arm prospective cohort | This prospective study is designed to evaluate the negative predictive value of LymphPET and to verify whether SLNB can be spared in patients with negative preoperative axillary assessment. | No | 1528 | NA | Distant and locoregional disease free survival | 2019 | Recruiting |
| **OMISSION OF SLNB IN N1 PATIENTS** | | | | | | | | | | | |
| **Name** | **Trial registry ID** | **Primary location** | **Multi-centre** | **Design** | **Research question** | **Biomarker stratified** | **Planned sample size** | **Final sample size** | **Primary outcome** | **Start year** | **Status** |
| ShandongCHI-13 | NCT04568941 | China | Yes | RCT | RCT of Comparison of Tumour Biopsy Method of Preoperative Vacuum-Assisted, Core Needle Versus Intraoperative Excisional Biopsy for SLNB in Breast Cancer with regards to node identification rate including false negative rate. | No | 600 | NR | SLN positive identification rate | 2020 | Unknown |
| **OMISSION OF ALND in SLNB+ PATIENTS** | | | | | | | | | | | |
| **Name** | **Trial registry ID** | **Primary location** | **Multi-centre** | **Design** | **Research question** | **Biomarker stratified** | **Planned sample size** | **Final sample size** | **Primary outcome** | **Start year** | **Status** |
| POSNOC | ISRCTN54765244 | Uk | Yes | RCT | Can women with breast cancer and 1 or 2 macrometastases, receive adjuvant therapy alone and it be non-inferior to adjuvant therapy plus axillary treatment, in terms of 5-year axillary recurrence? | No | 1900 | 1900 | Axillary recurrence | 2014 | Follow up |
| SERC | NCT01717131 | France | Yes | RCT | Is omission of completion ALND non-inferior to cALND for patients with an involved sentinel node? | No | 3000 | NA | Disease free survival | 2012 | Recruiting |
| BOOG 2013-07 | NCT02112682 | Netherlands | Yes | RCT | To determine whether omitting completion axillary treatment is not inferior to the current axillary treatment regimen in SLN+ breast cancer patients undergoing a mastectomy, in terms of regional recurrence rate. | No | 878 | 52 | Regional recurrence rate | 2014 | Terminated |
| SENOMAC | NCT02240472 | Sweden | Yes | RCT | Will refraining from ALND in breast cancer patients with 1-2 sentinel nodes with macrometastasis not worsen breast cancer-specific survival by more than a maximum of 2.5% after 5 years? | No | 3700 | 2700 | Breast cancer specific survival | 2015 | Follow up |
| SINODAR ONE | NCT05160324 | Italy | YES | RCT | To verify that the experimental treatment (omission of axillary lymph node intervention) in the presence of sentinel lymph node metastases does not lead to a significant worsening in survival or in the risk of locoregional recurrence compared to ALND | No | 2000 | 889 | Overall survival | 2015 | Terminated |
| SENOMIC | NCT02049632 | Sweden | Yes | Single-arm prospective cohort | What are the survival and axillary relapse rates in breast cancer patients with sentinel node micrometastases who have not undergone completion axillary clearance? | No | 452 | 805 | Disease free survival | 2013 | Complete |
| **OMISSION OF BREAST RADIOTHERAPY IN LOW/VERY LOW-RISK EARLY BREAST CANCER** | | | | | | | | | | | |
| **Name** | **Trial registry ID** | **Primary location** | **Multi-centre** | **Design** | **Research question** | **Biomarker stratified** | **Planned sample size** | **Final sample size** | **Primary outcome** | **Start year** | **Status** |
| PRIME II | ISRCTN95889329 | UK | Yes | RCT | To assess the role of post-operative breast RT in women aged 65 or older, with low risk breast cancer treated by BCS and adjuvant RT: in particular to estimate the difference in local recurrence rates between patients treated +/- RT. | No | 1294 | 1324 | Local breast cancer recurrence | 2003 | Complete |
| NATURAL | NCT03646955 | Denmark | Yes | RCT | Can PBI be safely omitted in selected low risk breast cancer patients without causing unacceptable risk of local failure? | No | 926 | NA | Invasive local recurrence | 2018 | Recruiting |
| EUROPA | NCT04134598 | Italy | Ns | RCT | In low-risk early stage patients ≥70 years, is exclusive radiation therapy (RT) approach or exclusive ET following BCS superior in terms of HRQoL and recurrence? | No | 926 | NA | IBTR and HRQoL | 2021 | Recruiting |
| EXPERT | NCT02889874 | Australia | Yes | RCT | To evaluate RT versus observation following BCS and planned ET in patients with stage I breast cancer of luminal A subtype defined using the Prosigna (PAM50) Assay. | Yes | 1167 | NA | Local recurrence rate | 2017 | Recruiting |
| DEBRA | NCT04852887 | USA | Yes | RCT | Evaluation of whether BCS and ET results in a non-inferior rate of invasive or non-invasive IBTR compared to BCS with breast radiation and ET | Yes | 1670 | NA | % ipsilateral breast tumour recurrence free | 2021 | Recruiting |
| HERO | NCT05705401 | USA | Yes | RCT | Comparison of RFI among patients with early-stage, low risk HER2+ breast cancer who undergo BCS and receive HER2-directed therapy and are randomized not to receive adjuvant breast RT versus those who are randomized to receive adjuvant RT per the current standard of care. | No | 1300 | NA | Recurrence free interval | 2023 | Recruiting |
| PROSPECT | ACTRN12610000810011 | Australia | Yes | 2-arm prospective cohort | To determine whether a combination of MRI and pathology findings can identify women with truly localised breast cancer who can safely omit radiotherapy. | No | 200 | 201 | Ipsilateral invasive recurrence rate | 2011 | Follow up |
| PRIMETIME | ISRCTN41579286 | UK | Yes | 2-arm prospective cohort | To obtain high-quality, practice-changing, clinical evidence supporting the safe avoidance of RT for a highly selected subgroup of breast cancer patients, who are deemed to be at such low risk of local relapse that the benefits associated with RT are unlikely to outweigh known risks. | Yes | 2400 | 2400 | Ipsilateral breast local relapse rate | 2015 | Follow up |
| LUMINA | NCT01791829 | Canada | Yes | Single-arm prospective cohort | To prospectively evaluate the usefulness of molecular definition of breast cancer in the identification of patients at very low risk for local recurrence after BCS when they were treated with ET without radiotherapy. | Yes | 500 | 500 | Ipsilateral breast tumour recurrence | 2013 | Complete |
| IDEA | NCT02400190 | USA | Yes | Single-arm prospective cohort | Can younger postmenopausal patients be successfully treated without radiotherapy using patient selection based on a commonly used genomic assay in addition to classic clinicopathologic selection factors? | Yes | 200 | 200 | Loco-regional recurrence | 2015 | Follow up |
| PRECISION | NCT02653755 | USA | Yes | 2-arm prospective cohort | To determine whether certain patients between the ages of 50 and 75 can safely omit RT after having a lumpectomy. using standard laboratory tests along with the Prosigna | Yes | 1380 | 672 | Local regional recurrence | 2016 | Follow up |
| TOP-1 | NL5983 | Netherlands | NS | Single-arm prospective cohort | Can RT be safely omitted after BCS in elderly patients at low risk of developing a LR? | No | 1182 | 1182 | Local recurrence rate | 2016 | Follow up |
| **PARTIAL BREAST IRRADIATION (PBI) IN EARLY BREAST CANCER** | | | | | | | | | | | |
| **Name** | **Trial registry ID** | **Primary location** | **Multi-centre** | **Design** | **Research question** | **Biomarker stratified** | **Planned sample size** | **Final sample size** | **Primary outcome** | **Start year** | **Status** |
| B39 RTOG 0413 | NCT00103181 | USA | Yes | RCT | A comparison of PBI and WBI following lumpectomy for patients with early-stage breast cancer to determine if PBI provided equivalent local tumour control. | No | 4214 | 4216 | In breast tumour recurrence | 2005 | Complete |
| COSMOPOLITAN | NCT03838419 | Germany | Yes | RCT | To analyze and contrast the intensity of fatigue in early-stage breast cancer treated with WBI or PBI after BCS. | No | 202 | NA | Fatigue score (FACIT) | 2019 | Recruiting |
| DBCG PBI | NCT00892814 | Denmark | Yes | RCT | Is moderately hypofractionated adjuvant external-beam PBI safe for patients with a low risk of recurrence? | No | 628 | 865 | Grade 2-3 breast induration | 2009 | Follow up |
| HYPAB | NCT02375048 | Italy | No | RCT | To report preliminary data of a randomized phase III trial comparing hypofractionated whole breast irradiation (HWBI) and PBI using volumetric modulated arc therapy (VMAT). | No | 700 | 172 | Cosmesis | 2015 | Terminated |
| IRMA | NCT01803958 | Italy | Yes | RCT | To evaluate whether partial hypofractionated and accelerated irradiation of the sole surgical cavity, in patients suffering from breast cancer with low risk of local recurrence and who undergo BCS, is not inferior to postoperative irradiation with conventional fractionation of the entire breast as regards local control. | No | 3302 | 3302 | Ipsilateral breast tumour recurrence | 2007 | Complete |
| LAPIDARY | NCT04669873 | Brazil | No | RCT | To evaluate the viability and safety of PBI, in 5 fractions, compared to radiotherapy regimens of the whole breast in 15 and 5 fractions, in patients with breast cancer, in initial stage, who underwent conservative surgery. | No | 36 | NA | Rate of local recurrence | 2021 | Recruiting |
| NV19-03-00354 | NCT06007118 | Czech Rep | No | RCT | To compare the targeted external beam PBI with commonly used accelerated WBI in terms of feasibility, safety, tolerance, and cosmetic effects. | No | 85 | 84 | Toxicity, cosmesis, feasibility, tolerance, and safety | 2019 | Complete |
| OPAL | NCT03077841 | USA | Yes | RCT | Does treating only the part of the breast where the cancer started lead to fewer side effects than standard treatment consisting of whole breast irradiation? | No | 928 | NA | Risk of ≥grade 2 toxicity | 2017 | Follow up |
| RAPID | NCT00282035 | Canada | Yes | RCT | To find out if external beam PBI delivered in 1 week was non-inferior to whole breast irradiation with respect to preventing local recurrence after BCS | No | 2128 | 2135 | Ipsilateral breast tumour recurrence | 2006 | Complete |
| SHARE | NCT01247233 | France | Yes | RCT | The objective is to compare the effectiveness and safety of PBI compared with whole breast irradiation. | No | 2796 | 1006 | Rate of local recurrence | 2010 | Follow up |
| ELDERLY APBI | NL2791 | Netherlands | Yes | 2-arm prospective cohort | To determine if local control and toxicity in breast conserving therapy using PBI is not inferior to classical 50 Gy irradiation | No | 710 | 596 | Loco-regional tumour control | 2011 | Complete |
| PAPBI | NCT01024582 | Netherlands | No | Single-arm prospective cohort | To study preoperative Accelerated Partial Breast Irradiation (PAPBI) in low risk patients >60 years with unifocal nonlobular adenocarcinoma and negative sentinel node in a multi center feasibility study with assessment of local recurrence. | No | 120 | 133 | Local recurrence | 2009 | Complete |
| IRB–13807 | NCT00185744 | USA | No | 2-arm prospective cohort | To determine whether an accelerated course of RT delivered to the lumpectomy cavity plus margin using IORT as a single dose, intracavitary brachytherapy with the MammoSite device over 5 days, partial breast 3-D CRT in 5 days, or stereotactic PBI over 4 days is a feasible and safe alternative to a six and a half week course of whole breast RT. | No | NS | 400 | In breast tumour recurrence | 2002 | Follow up |
| **INTRAOPERATIVE RADIOTHERAPY (IORT)** | | | | | | | | | | | |
| **Name** | **Trial registry ID** | **Primary location** | **Multi-centre** | **Design** | **Research question** | **Biomarker stratified** | **Planned sample size** | **Final sample size** | **Primary outcome** | **Start year** | **Status** |
| TARGIT-A | ISRCTN34086741 | UK | Yes | RCT | To determine whether risk adapted IORT, delivered as a single dose during lumpectomy, can effectively replace postoperative whole breast external beam radiotherapy for early breast cancer. | No | 2232 | 2298 | Local recurrence rate | 2000 | Complete |
| TARGIT E | NCT01299987 | Germany | Yes | Single-arm prospective cohort | To investigate the efficacy of a single IORT treatment within elderly low risk patients (≥ 70 years, cT1, cN0, cM0, invasive-ductal) which is followed by WBRT only when risk factors are present. | No | 265 | 541 | Local relapse rate | 2011 | Follow up |
| TARGIT C | NCT02290782 | Germany | Yes | Single-arm prospective cohort | To confirm the efficacy of a single dose of IORT in a well selected group of patients with small breast cancer and absence of risk factors. | No | 387 | 387 | Local relapse rate | 2014 | Follow up |
| N/A | UMIN000003578 | Japan | No | Single-arm prospective cohort | What is the efficacy and safety of IORT in this patient group? | No | 140 | 142 | Ipsilateral local recurrence rate | 2010 | Complete |
| IORT2 | NCT02400658 | USA | Yes | Single-arm prospective cohort | To evaluate an investigational way to provide radiation therapy to treat early-stage breast cancer at the time of surgery. | No | 260 | 358 | Local breast tumour recurrence | 2015 | Follow up |
| CTPR-0009 | NCT01644669 | USA | Yes | Single-arm prospective cohort | To assess the safety and efficacy of the Xoft Axxent eBx System when used for single-fraction IORT in early stage breast cancer. | No | 1000 | 1200 | Ipsilateral breast tumour recurrence | 2012 | Follow up |
| **INTERSTITIAL BRACHYTHERAPY IN EARLY BREAST CANCER** | | | | | | | | | | | |
| **Name** | **Trial registry ID** | **Primary location** | **Multi-centre** | **Design** | **Research question** | **Biomarker stratified** | **Planned sample size** | **Final sample size** | **Primary outcome** | **Start year** | **Status** |
| GEC-ESTRO APBI | NCT00402519 | Germany | Yes | RCT | To assess the role of interstitial brachytherapy alone compared to WBI in a defined low-risk group of invasive breast cancer or DCIS concerning local failure (to affirm the hypothesis that local control rates in each arm are equivalent. | No | 1166 | 1328 | Ipsilateral local recurrence | 2004 | Complete |
| TRIUMPH-T | NCT02526498 | USA | Yes | Single-arm prospective cohort | To determine the toxicity rate with shorter courses of PBI delivered with a breast brachytherapy applicator. | No | 200 | 200 | Grade 2 or worse toxicity event(s) | 2015 | Complete |
| SiFEBI | NCT01727011 | France | No | Single-arm prospective cohort | To evaluate the clinical outcomes of a very-accelerated partial breast irradiation (vAP- BI) in the elderly based on a single fraction of multicatheter interstitial high-dose rate brachytherapy (MIB). | No | 25 | 26 | Acute toxicity | 2012 | Complete |
| **OMISSION OF PMRT IN INTERMEDIATE RISK PATIENTS (N1)** | | | | | | | | | | | |
| **Name** | **Trial registry ID** | **Primary location** | **Multi-centre** | **Design** | **Research question** | **Biomarker stratified** | **Planned sample size** | **Final sample size** | **Primary outcome** | **Start year** | **Status** |
| SUPREMO | ISRCTN61145589 | UK | Yes | RCT | To determine the effect of ipsilateral chest wall irradiation following mastectomy and axillary clearance versus no irradiation for women with operable breast cancer at 'intermediate risk' of loco-regional recurrence. | No | 3700 | 1688 | Overall survival | 2005 | Complete |
| **OMISSION OF AXILLARY/REGIONAL RT IN SLNB+ PATIENTS** | | | | | | | | | | | |
| **Name** | **Trial registry ID** | **Primary location** | **Multi-centre** | **Design** | **Research question** | **Biomarker stratified** | **Planned sample size** | **Final sample size** | **Primary outcome** | **Start year** | **Status** |
| OPTIMAL | NCT02335957 | Spain | Yes | RCT | To show the non-inferiority of the incidental irradiation, as compared to intentional irradiation of the axillary nodes, in terms of 5-years DFS of early-stage breast cancer patients with limited affectation of sentinel node assessed by OSNA (250 to 15,000 copies/uL), treated with BCS without ALND | No | 1422 | 489 | Disease free survival | 2015 | Terminated |
| T-REX | NCT05634889 | Sweden | Yes | RCT | To evaluate whether regional radiotherapy may safely be omitted in patients with limited lymph node metastasis and an estimated low risk of locoregional recurrence. | No | 1350 | NA | Recurrence free survival | 2023 | Recruiting |
| **OMISSION OF REGIONAL NODAL IRRADIATION (RNI)** | | | | | | | | | | | |
| **Name** | **Trial registry ID** | **Primary location** | **Multi-centre** | **Design** | **Research question** | **Biomarker stratified** | **Planned sample size** | **Final sample size** | **Primary outcome** | **Start year** | **Status** |
| KROG 1701 | NCT03269981 | South Korea | Yes | RCT | To evaluate the impact of elective RNI on N1 breast cancer patients receiving post-lumpectomy radiotherapy and anthracycline plus taxane (AT)-based chemotherapy. | No | 1926 | 827 | Disease free survival | 2017 | Follow up |
| PORT-N1 | NCT05440149 | South Korea | Yes | RCT | To evaluate the feasibility of post-operative radiotherapy (PORT) de-escalation in pN1 breast cancer patients by establishing indications for RNI in patients receiving BCS, and for PMRT in patients receiving a mastectomy. | No | 1106 | NA | Disease free survival | 2022 | Recruiting |
| TAILOR RT | NCT03488693 | Canada | Yes | RCT | To compare the effects on low-risk breast cancer (N1) receiving usual care that includes regional radiation therapy, with receiving no regional radiation therapy. | Yes | 2140 | NA | Breast cancer recurrence free interval | 2018 | Recruiting |
| **OMISSION OF IMC RT IN EARLY-STAGE INTERMEDIATE RISK (N1) PATIENTS** | | | | | | | | | | | |
| **Name** | **Trial registry ID** | **Primary location** | **Multi-centre** | **Design** | **Research question** | **Biomarker stratified** | **Planned sample size** | **Final sample size** | **Primary outcome** | **Start year** | **Status** |
| IMNI PRECISION | NCT04517266 | China | No | RCT | Evaluation of omitting IMC RT among early stage intermediate risk (N1) breast cancer patients according to clinical-genomic model with regards to event free survival. | Yes | 214 | NA | Event free survival | 2021 | Recruiting |
| **pCR AFTER SINGLE DOSE OF MRI GUIDED PRE-OP PBRT IN LOW-RISK BREAST CANCERS** | | | | | | | | | | | |
| **Name** | **Trial registry ID** | **Primary location** | **Multi-centre** | **Design** | **Research question** | **Biomarker stratified** | **Planned sample size** | **Final sample size** | **Primary outcome** | **Start year** | **Status** |
| ABLATIVE-2 | NCT05350722 | Netherlands | Yes | Single-arm prospective cohort | What is the rate of pCR after low risk breast cancer patients receive a single dose of MRI guided pre-operative partial breast irradiation? | No | 100 | NA | % achieving pCR | 2022 | Recruiting |

A-pCR – axillary complete pathological response; ALND – axillary lymph node dissection; ART – axillary radiotherapy; BC – breast cancer; BCS – breast conserving surgery; DFS – disease free survival; ER – oestrogen receptor; ET – endocrine therapy; Gy – Gray; HRQoL – health-related quality of life; IBTR – ipsilateral breast tumour recurrence; IMC – internal mammary chain; LR – local recurrence; MRI – magnetic resonance imaging; NST – neoadjuvant systemic therapy; OSNA – one-step nucleic acid amplification; PBI – partial breast irradiation; pCR – pathological complete response; PET – positron-emission tomography; PMRT – post mastectomy radiotherapy; RCT – randomised controlled trial; RFA – radiofrequency ablation; RFI – recurrence free interval; RFS – recurrence free survival; RNI – regional nodal irradiation; RR – regional recurrence; RT – radiotherapy; SLN – sentinel lymph node; SLNB – sentinel lymph node biopsy; TAS – targeted axillary surgery; TNBC – triple negative breast cancer; US – ultrasound; VAB – vacuum-assisted biopsy; VAE – vacuum-assisted excision

**Supplementary Table 3: Study designs and hypothesis testing**

| **Cohort studies** | | | | | | |
| --- | --- | --- | --- | --- | --- | --- |
| **Outcome type** | **Name of study** | **Trial registry ID number** | **Treatment de-escalation evaluated** | **Power** | **Designed to evaluate** | **Primary outcome measure** |
| **Locoregional recurrence endpoints** | EUBREAST-01 | NCT04101851 | Omission of axillary staging in cN0 patients after NST | 95 | Pre-defined acceptable threshold  ARFS ≥98.5% but ≥96.5% <98.5% tolerated | Axillary recurrence free survival |
|  | ICE 3 | NCT02200705 | Omission of surgery in small low risk tumours | 99 | Pre-defined acceptable threshold - IBTR of 2.5% or less | In breast tumour recurrence |
|  | TTUHSC IRB #L18-100 | N/A | Omission of surgery in small low risk tumours | NS | Pre-defined acceptable threshold -Recurrence rate ≤2% | Local recurrence |
|  | PRIMETIME | ISRCTN41579286 | Omission of breast RT in low risk patients | NS | Pre-defined acceptable threshold - Relapse rate of ≤4% | Ipsilateral breast local relapse rate |
|  | PAPBI | NCT01024582 | PBI | NS | Pre-defined acceptable threshold - Local recurrence ≤4% | Local recurrence |
|  | IDEA | NCT02400190 | Omission of breast RT in low risk patients | NS | Pre-defined acceptable threshold - Recurrence rate ≤4% | Loco-regional recurrence |
|  | TARGIT C | NCT02290782 | IORT | 90 | Pre-defined acceptable threshold - Local relapse rate between 1.4% and 4% | Local relapse rate |
|  | TARGIT E | NCT01299987 | IORT | 90 | Pre-defined acceptable threshold - Relapse margin of 4.5% at 7.5 years follow up | Local relapse rate |
|  | LUMINA | NCT01791829 | Omission of breast RT in low risk patients | 90 | Pre-defined acceptable threshold - upper boundary of 2-sided 90% CI for IBTR <5% | Ipsilateral breast tumour recurrence |
|  | PROSPECT | ACTRN12610000810011 | Omission of breast RT in low risk patients | NS | Pre-defined acceptable threshold - IIRR of 5% | Ipsilateral invasive recurrence |
|  | PRECISION | NCT02653755 | Omission of breast RT in low risk patients | 90 | Pre-defined acceptable threshold - LRR of 5% | Local regional recurrence |
|  | DESCARTES | NCT05416164 | Omission of breast RT in cN0 patients with pCR post NST | 80 | Pre-defined acceptable threshold - LR rate <6% | Local recurrence |
|  | N/A | UMIN000003578 | IORT | NS | Pre-defined acceptable threshold - Recurrence rate ≤6% | Ipsilateral local recurrence rate |
|  | ALLIANCE Z11102 | NCT01556243 | Omission of mastectomy in patients with multiple ipsilateral primary breast cancers | NS | Pre-defined acceptable threshold - Local recurrence <8% | Local recurrence |
|  | ELDERLY APBI | NL2791 | PBI | NS | Pre-defined acceptable threshold - LR >10% at 5 years | Loco-regional tumour control |
| **Composite survival-based endpoints** | SENOMIC | NCT02049632 | Omission of ALND in SLNB+(micro) patients | 80 | Pre-defined acceptable threshold - DFS ≥80% | Disease free survival |
|  | OPTIMIST | NCT05505357 | Omission of breast surgery in patients with pCR post NST | 80 | Pre-defined acceptable threshold - 5% | Disease free survival |
|  | NEONOD2 | NCT04019678 | Omission of ALND in patients with ypN+ disease post NST | 80 | Pre-defined acceptable threshold - DFS <10% | Disease free survival |
|  | ASLAN | NCT04993625 | Omission of SNB in cN0 patients with pCR post NST | NS | Pre-defined acceptable threshold - RFS ≥84% | Recurrence free survival |
| **Technical endpoints** | GANEA3 | NCT03630913 | Omission of ALND in patients cN1 to cN0 post NST | 80 | Pre-defined acceptable threshold - Reduction in FNR from 14% to 1% | False negative rate |
|  | FIRST | NCT05398497 | Omission of surgery in small low risk tumours | 95 | Pre-defined acceptable threshold - Success rate >70% | Absence of residual disease |
|  | BR-003 | NCT03463954 | Omission of surgery in small low risk tumours | NS | Pre-defined acceptable threshold - Tumour ablation rate >87.85% | Absence of residual disease |
|  | PetrovRIO | NCT04293796 | Omission of breast surgery in patients with pCR post NST | NS | Pre-defined acceptable threshold - FNR <15% | False negative rate |
|  | ABLATIVE-2 | NCT05350722 | pCR after MRI guided pre-op RT in low-risk patients | NS | Pre-defined acceptable threshold - >40% achieve pCR | % achieving pCR |
| **Toxicity** | TRIUMPH-T | NCT02526498 | Brachytherapy | NS | Pre-defined acceptable threshold - Toxicity events <10% | Grade 2 or worse toxicity event(s) |
| **Randomised controlled trials** | | | | | | |
| **Outcome type** | **Name of study** | **Trial registry ID number** | **Treatment de-escalation evaluated** | **Power** | **Designed to evaluate** | **Primary outcome measure** |
| **Locoregional recurrence endpoints** | B39 RTOG 0413 | NCT00103181 | PBI | 85 | Equivalence - Margin of 50% increase in RR | In breast tumour recurrence |
|  | SHARE | NCT01247233 | PBI | 80 | Non-inferiority - HR does not exceed 1.6 | Rate of local recurrence |
|  | ALLIANCE A011202 | NCT01901094 | Omission of ALND in ypN+ patients post NST | 87 | Non-inferiority - HR in intervention arm of 0.0923 | Invasive breast cancer recurrence free interval |
|  | TAILOR RT | NCT03488693 | Omission of RNI in low risk N1 patients | 87 | Non-inferiority - HR does not exceed 1.4 | Breast cancer recurrence free interval |
|  | IRMA | NCT01803958 | PBI | 90 | Non-inferiority - HR upper CI not >1.5 | Ipsilateral breast tumour recurrence |
|  | RAPID | NCT00282035 | PBI | 85 | Non-inferiority - NIM 1.5% | Ipsilateral breast tumour recurrence |
|  | POSNOC | ISRCTN54765244 | Omission of axillary treatment in SLNB+ patients | 80 | Non-inferiority - NIM 2% | Axillary recurrence |
|  | TARGIT-A | ISRCTN34086741 | IORT | 80 | Non-inferiority - NIM 2.5% | Local recurrence rate |
|  | PRIME II | ISRCTN95889329 | Omission of breast RT in low-risk patients | 80 | Non-inferiority - NIM 3% | Local breast cancer recurrence |
|  | GEC-ESTRO APBI | NCT00402519 | PBI | 80 | Non-inferiority - NIM 3% | Ipsilateral local recurrence |
|  | NATURAL | NCT03646955 | Omission of breast RT in low risk patients | 80 | Non-inferiority - NIM 3% | Invasive local recurrence |
|  | EUROPA | NCT04134598 | Omission of breast RT in low risk patients | 80 | Non-inferiority - NIM 3% | Ipsilateral breast tumour recurrence and HRQoL |
|  | DEBRA | NCT04852887 | Omission of breast RT in low risk patients | 80 | Non-inferiority - NIM 4% | % ipsilateral breast tumour recurrence free |
|  | BOOG 2013-07 | NCT02112682 | Omission of axillary treatment in SLNB+ patients | 80 | Non-inferiority - NIM 5% | Regional recurrence rate |
|  | BOOG 2013-08 | NCT02271828 | Omission of SLNB in cN0 patients | 80 | Non-inferiority - NIM 5% | Regional recurrence rate |
| **Composite survival-based endpoints** | SERC | NCT01717131 | Omission of ALND in SLNB+ patients | 85 | Non-inferiority - NIM 1.25% | Disease free survival |
|  | ADARNAT | NCT04889924 | ALND vs ART in SLNB+ patients post SNT | 80 | Non-inferiority - NIM 2% | Disease free survival |
|  | SOUND | NCT02167490 | Omission of SLNB in cN0 patients | 80 | Non-inferiority - NIM 2.5% | Distant disease free survival |
|  | SENOMAC | NCT02240472 | Omission of ALND in SLNB+ patients | 80 | Non-inferiority - NIM 2.5% | Breast cancer specific survival |
|  | INSEMA | NCT02466737 | Omission of SLNB in cN0 patients | NS | Non-inferiority - NIM 3% | Invasive disease free survival |
|  | ATNEC | ISRCTN36585784 | Omission of axillary treatment in patients converting to cN+ to ypN0 post NST | 85 | Non-inferiority - NIM 3.5% | DFS and rates of patient reported lymphoedema |
|  | EXPERT | NCT02889874 | Omission of breast RT in low risk patients | NS | Non-inferiority - NIM 4% | Local recurrence rate |
|  | HERO | NCT05705401 | Omission of breast RT in low risk patients | NS | Non-inferiority - NIM 3.6% | Recurrence free interval |
|  | SINODAR ONE | NCT05160324 | Omission of ALND in SLNB+ patients | 80 | Non-inferiority - NIM 4% | Overall survival |
|  | T-REX | NCT05634889 | Omission of axillary/regional RT in SLNB+ patients | 80 | Non-inferiority - NIM 4.5% | Recurrence free survival |
|  | OPTIMAL | NCT02335957 | Omission of axillary/regional RT in SLNB+ patients | 80 | Non-inferiority - NIM 5% | Disease free survival |
|  | TAXIS | NCT03513614 | Omission of ALND in cN+ patients | 80 | Non-inferiority - NIM 5% | Disease free survival |
|  | VENUS | NCT05315154 | Omission of SLNB in cN0 patients | 80 | Non-inferiority - NIM 5% | Disease free survival |
|  | PORT-N1 | NCT05440149 | Omission of RNI | 80 | Non-inferiority - NIM 5% | Disease free survival |
|  | SUPREMO | ISRCTN61145589 | Omission of PMRT in intermediate risk patients | 80 | Non-inferiority - NIM 7% | Overall survival |
|  | NAUTILUS | NCT04303715 | Omission of SLNB in cN0 patients | 80 | Non-inferiority - NIM 5% | Invasive disease-free survival |
|  | IMNI PRECISION | NCT04517266 | Omission of IMC radiation in N1 patients | 80 | Non-inferiority - NIM 10% | Event free survival |
| **Toxicity or morbidity related endpoints** | DBCG PBI | NCT00892814 | PBI | 80 | Non-inferiority - NIM 10% | Grade 2-3 breast induration |
|  | NV19-03-00354 | NCT06007118 | PBI | 90 | Non-inferiority - NIM 10% | Toxicity, cosmesis, feasibility, tolerance, and safety |
| **Technical endpoints** | SMALL | ISRCTN12240119 | Omission of surgery in small low risk tumours | 90 | Non-inferiority - NIM10% | Re-excision rates and local recurrence rate |
|  | THERMAC | NL9205 | Omission of surgery in small low risk tumours | 80 | Superiority - Efficacy rate >85% and difference between arms of 10% | Absence of residual disease |
| **Health-related quality of life endpoints** | COSMOPOLITAN | NCT03838419 | PBI | 90 | Superiority - 6 points FACIT | Fatigue score (FACIT) |
| **Cosmesis** | HYPAB | NCT02375048 | PBI | 80 | Superiority - 10% improvement in cosmesis | Cosmesis |

ALND – axillary lymph node dissection; PBI – accelerated partial breast irradiation, ARFS – axillary recurrence free survival; ART – axillary radiotherapy; BC – breast cancer; BCS – breast conserving surgery; CI – confidence interval; DFS – disease free survival; ER – oestrogen receptor; ET – endocrine therapy; FNR – false negative rate; Gy – Gray; HR – hazard ratio; HRQoL – health-related quality of life; IBTR – ipsilateral breast tumour recurrence; IIR – ipsilateral invasive recurrence; IMC – internal mammary chain; IORT – intraoperative radiotherapy; LR – local recurrence; MRI – magnetic resonance imaging; NIM – non-inferiority margin; NST – neoadjuvant systemic therapy; PBI – partial breast irradiation; pCR – pathological complete response; PET – positron-emission tomography; PMRT – post mastectomy radiotherapy; RCT – randomised controlled trial; RFA – radiofrequency ablation; RFI – recurrence free interval; RFS – recurrence free survival; RNI – regional nodal irradiation; RR – regional recurrence; RT – radiotherapy; SLN – sentinel lymph node; SLNB – sentinel lymph node biopsy

**Supplementary Table 4: Search strategies**

| **ONLINE DATABASE SEARCHES** | | |
| --- | --- | --- |
| **OVID MEDLINE Search Strategy - 16/01/2024** | | |
|  | **Search terms including OVID commands** | **Number of results** |
| **1** | exp Breast Neoplasms/ | 348957 |
| **2** | exp Axilla/ | 14795 |
| **3** | exp Carcinoma, Intraductal, Non-infiltrating/ | 11339 |
| **4** | 1 or 2 or 3 | 356501 |
| **5** | exp Mastectomy, Segmental/ | 10343 |
| **6** | breast conserving surgery.mp. | 7046 |
| **7** | lumpectomy.mp. | 3905 |
| **8** | exp Sentinel Lymph Node Biopsy/ or exp Lymph Node Excision/ | 55600 |
| **9** | exp Radiotherapy/ or exp Radiotherapy, Adjuvant/ | 209688 |
| **10** | exp Brachytherapy/ | 22019 |
| **11** | intraoperative radiotherapy.mp. | 1354 |
| **12** | partial breast irradiation.mp. | 1567 |
| **13** | vacuum assisted.mp. | 4335 |
| **14** | ablation.mp. | 131023 |
| **15** | 5 or 6 or 7 or 8 or 9 or 10 or 11 or 12 or 13 or 14 | 403838 |
| **16** | de-escalation.mp. | 3762 |
| **17** | non-inferiority.mp. | 8363 |
| **18** | exp Therapeutic Equivalency/ or Equivalence Trial/ | 9448 |
| **19** | 16 or 17 or 18 | 21010 |
| **20** | 4 and 15 and 19 | 276 |
| **21** | 20 and 2019:2023.(sa_year). | **209** |
| **OVID EMBASE search strategy: 16/01/2024** | | |
| **1** | exp Breast Neoplasms/ | 663243 |
| **2** | exp Axilla/ | 12544 |
| **3** | exp Carcinoma, Intraductal, Non-infiltrating/ | 2752 |
| **4** | 1 or 2 or 3 | 671625 |
| **5** | exp Mastectomy, Segmental/ | 23520 |
| **6** | breast conserving surgery.mp. | 13279 |
| **7** | lumpectomy.mp. | 10127 |
| **8** | exp Sentinel Lymph Node Biopsy/ or exp Lymph Node Excision/ | 99311 |
| **9** | exp Radiotherapy/ or exp Radiotherapy, Adjuvant/ | 665479 |
| **10** | exp Brachytherapy/ | 57587 |
| **11** | intraoperative radiotherapy.mp. | 3685 |
| **12** | partial breast irradiation.mp. | 3420 |
| **13** | vacuum assisted.mp. | 14886 |
| **14** | ablation.mp. | 216984 |
| **15** | 5 or 6 or 7 or 8 or 9 or 10 or 11 or 12 or 13 or 14 | 975373 |
| **16** | de-escalation.mp. | 7150 |
| **17** | non-inferiority.mp. | 16327 |
| **18** | exp Therapeutic Equivalency/ or Equivalence Trial/ | 2373 |
| **19** | 16 or 17 or 18 | 25711 |
| **20** | 4 and 15 and 19 | 881 |
| **21** | 20 and 2019:2023.(sa_year). | **659** |
| **COCHRANE LIBRARY search strategy: 16/01/2024** | | |
| . | ‘Breast cancer’ OR ‘axillary’ OR ‘sentinel node’ OR ‘carcinoma in situ’ AND (‘surgery’ OR ‘radiation’ OR ‘biopsy’ OR ‘ablate’ OR ‘vacuum’ OR ‘margin’) AND (‘non-inferior’ OR ‘de-escalate’ OR ‘equivalence’ OR ‘omit’ OR ‘versus’). Search terms within title, abstract, or key word. Word variations searched. 2019-2023 in English | **1442** |
| **TRIAL REGISTRY SEARCH STRATEGIES** | | |
| **CLINICALTRIALS.GOV search strategy: 16/01/2024** | | |
|  | Condition/disease: ‘breast cancer’. Other terms: ‘de-escalation’ OR ‘non-inferior’ OR ‘ablation’ OR ‘vacuum’ OR ‘omission’ OR ‘radiotherapy’ OR ‘surgery’ OR ‘margin’. Filter to include ‘not yet recruiting’, ‘recruiting’, ‘active trials’, ‘phase II’, ‘phase III’, ‘adult females including elderly’, and ‘interventional’. | **1340** |
| **DUTCH TRIALS REGISTRY search strategy 16/01/2024** | | |
|  | Search heading: ‘breast cancer’ (N = 493)  Search filter: registered from 2013 and recruiting (N = 143)  Search filter: trial type interventional | **90** |
| **ISRCTN search strategy 16/01/2024** | | |
|  | Condition: ‘breast cancer’; study status: ‘ongoing’ | **64** |
| **EU CLINICAL TRIALS REGISTRY search strategy 16/01/2024** | | |
|  | ‘Breast cancer surgery’ AND ‘breast cancer radiotherapy’  Phase II/III trials in adults currently ongoing | **187** |
